# Molecular insights into nilvadipine–hemoglobin interactions: conformational dynamics and binding mechanisms

**DOI:** 10.1039/d5ra04162g

**Published:** 2025-11-11

**Authors:** Mohd Shahnawaz Khan, Md. Tabish Rehman, Nojood Al-twaijry, Nouf Omar Alafaleq, Ibrahim Aldobiyan, Majed S. Alokail, Areej Ali Alzahrani, Mohammed Arshad, Mohammad F. AlAjmi

**Affiliations:** a Department of Biochemistry, College of Science, King Saud University Riyadh 11451 Saudi Arabia moskhan@ksu.edu.sa; b Department of Pharmacognosy, College of Pharmacy, King Saud University Riyadh 11451 Saudi Arabia; c Dental Health Department, College of Applied Medical Science, King Saud University Riyadh 11451 Saudi Arabia

## Abstract

Understanding the molecular basis of drug–protein interactions is essential for predicting pharmacokinetics and potential off-target effects. Here, we employ a combined experimental and computational approach to characterize the binding of Nilvadipine (a dihydropyridine calcium channel blocker) to hemoglobin (Hb). Using Soret band absorption and steady-state fluorescence spectroscopy across 298–310 K, we observed pronounced static quenching of Hb's intrinsic fluorescence, yielding Stern–Volmer constants (*K*_SV_) in the order of 10^4^ M^−1^ and 1 : 1 binding stoichiometry. Thermodynamic parameters derived from van't Hoff analysis (Δ*H*° > 0, Δ*S*° > 0, and Δ*G*° < 0) highlighted hydrophobic interactions as the primary driving force and confirmed the spontaneity of complex formation. Förster resonance energy transfer (FRET) measurements further positioned Nilvadipine at ∼3.0 nm from Hb's fluorophores, consistent with a static, ground-state complex. Molecular docking identified a preferential binding pose stabilized by hydrogen bonds with ASN68 and ASP64, hydrophobic contacts involving ALA82, LEU83, and LEU86, and interactions with the heme group, yielding a computed binding energy of −5.50 kcal mol^−1^ in close agreement with spectroscopically derived Δ*G*°. Over 100 ns of molecular dynamics (MD) simulations, the Hb–Nilvadipine complex remained structurally robust, with backbone RMSD values <0.2 nm, minor radius of gyration (*R*_g_) reduction, limited per-residue fluctuations (RMSF < 0.3 nm), and negligible changes in solvent-accessible surface area (SASA). Together, these data demonstrate that Nilvadipine forms a stable, hydrophobically driven complex with Hb without perturbing its global fold, suggesting that Hb may serve as a transient reservoir for the drug in circulation. This integrative study provides a detailed roadmap for interrogating small-molecule binding to blood proteins and offers insights valuable for drug delivery, safety assessment, and the design of Hb-based carriers.

## Abstract 2

Understanding the molecular basis of drug–protein interactions is essential for predicting pharmacokinetics and potential off-target effects.

## Graphical abstract

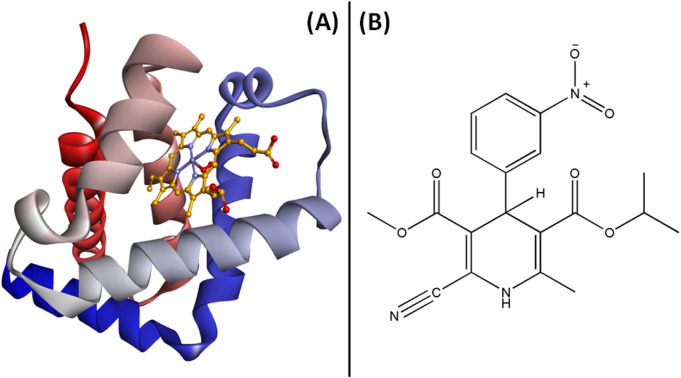

## 1. Introduction

In recent years, the study of protein–ligand interactions have gained considerable traction across the fields of biochemistry, molecular biology, pharmacology, and food science. These interactions offer valuable insights into the structural and functional dynamics of proteins, their roles in physiological and pathological processes, and their responses to xenobiotic compounds.^1^ In the health sector, protein–drug interaction studies are pivotal for drug discovery, delivery, and safety assessment, as they help determine pharmacokinetic properties such as bioavailability, metabolism, and off-target effects. In the food industry, understanding how bioactive compounds, food additives, or contaminants interact with dietary or endogenous proteins can influence nutritional quality, allergenicity, stability, and functional food design.^2^

Hemoglobin (Hb), the principal oxygen carrier in red blood cells, is a tetrameric metalloprotein composed of two α- and two β-globin chains, each harboring a heme prosthetic group with a central Fe^2+^ ion. This iron center reversibly binds O_2_, enabling efficient uptake in the lungs and delivery to peripheral tissues.^3^ Each globin chain adopts an eight-helix fold (helices A–H) that creates a hydrophobic heme pocket, and cooperative allosteric interactions among subunits fine-tune oxygen affinity, a feature essential for physiological function.^4^ Beyond gas transport, Hb is a prominent binding partner for a wide range of xenobiotics especially small-molecule drugs owing to its high abundance and multiple non-covalent interaction sites. The most notable binding regions are the hydrophobic cavities adjacent to the heme moieties and the inter-subunit interfaces. Within the heme pocket, ligands can engage in π–π stacking with the porphyrin ring, coordinate to the iron, or form hydrogen bonds with residues such as histidine, tyrosine, and phenylalanine. Additional peripheral sites on the protein surface permit van der Waals and electrostatic contacts with various ligands.^5^ Hb's defined structure, excellent solubility, and intrinsic absorbance make it an ideal model for probing ligand binding. Its adaptable binding pockets can accommodate diverse compounds ranging from cardiovascular drugs and antibiotics to flavonoids, vitamins, and food preservatives, which in turn can modify food product stability or influence the behavior of co-formulated nutraceuticals. Multiple spectroscopic and computational investigations have detailed these interactions. For example, fluorescence and UV-Vis studies showed that ibuprofen binds strongly to Hb and induces conformational shifts.^6,7^ Doxycycline was found to associate with Hb primarily through hydrogen bonds and hydrophobic interactions, potentially altering tertiary structure.^8^ Doxorubicin, an anthracycline chemotherapeutic, has also been reported to coordinate with the heme iron and disturb Hb's native conformation.^9^ Such binding events can affect drug distribution, provoke oxidative stress, or impair oxygen transport, highlighting the necessity of thoroughly characterizing drug–Hb interactions during pharmacokinetic and safety assessments.

Nilvadipine (Nil) is a dihydropyridine calcium channel blocker extensively prescribed for hypertension and evaluated for neuroprotective benefits owing to its ability to traverse the blood–brain barrier and favorably modulate cerebral perfusion.^10^ Its lipophilic nature, well-established safety profile, and clinical relevance make it an excellent probe for investigating drug–protein interactions with Hb, shedding light on systemic distribution and potential allosteric effects.^11^ Nilvadipine inhibits L-type voltage-dependent calcium channels by physically occluding the channel pore, thereby reducing Ca^2+^ influx into vascular smooth muscle and cardiomyocytes and producing antihypertensive effects. Beyond vascular modulation, Nilvadipine exhibits neuroprotective actions such as lowering amyloid-β (Aβ_40_ and Aβ_42_) production and enhancing Aβ clearance across the blood–brain barrier in Alzheimer's disease models.^12,13^ Clinical investigations have demonstrated that Nilvadipine increases hippocampal cerebral blood flow in hypertensive and Alzheimer's patients, indicating improved cerebrovascular regulation.^14^

In this study, we selected Hb as a representative protein to investigate how it interacts with Nilvadipine at the molecular level. By employing Soret band absorption, fluorescence spectroscopy and circular dichroism, we assessed how Nilvadipine binding affects Hb's fluorescence quenching and induces structural rearrangements. Complementary computational studies such as molecular docking and molecular dynamics simulation led to the identification of the preferred binding site of Nilvadipine, including any coordination to the heme iron or contacts with key amino acids such as histidine and phenylalanine.

## 2. Material and methods

### 2.1. Materials

Hemoglobin (Sigma-Aldrich (H7379), St. Louis, MO, USA) was dissolved in 50 mM phosphate buffer (pH 7.4) to yield a 5 mg per ml stock solution. For spectroscopic and binding assays, this stock was diluted to a working concentration of 0.2 mg ml^−1^. Nilvadipine (Sigma-Aldrich (SML0945), St. Louis, MO, USA) was prepared as a 10 mM stock in DMSO. All additional reagents were of analytical grade, and experiments were conducted using Milli-Q water.

### 2.2. Methods

#### 2.2.1. Fluorescence spectroscopy

Fluorescence experiments were carried out on a Jasco FP-750 spectrofluorometer (Tokyo, Japan) equipped with a Peltier-controlled thermostat (ETC-815) and a circulating water bath. Intrinsic emission spectra were collected at 298, 303, and 310 K using 1.0 cm quartz cuvettes, as reported previously.^15^ Samples were excited at 295 nm, and emission was monitored from 300 to 400 nm with both excitation and emission bandwidths set to 5 nm. Hb was held constant at 5 μM in each measurement, while Nilvadipine concentrations were titrated from 0 to 50 μM in a total volume of 1.0 ml. Fluorescence intensities were corrected for inner-filter effects^16^ according to the previously published protocol using eqn (1).

1 *F*_corr_ = *F*_obs_ × 10^(*A*_ex_+*A*_em_)/2^

where, *F*_corr_ and *F*_obs_ were the corrected and observed fluorescence intensities respectively. In addition, *A*_ex_, and *A*_em_ were the absorption of the drug at excitation (*λ*_ex_) and emission (*λ*_em_) wavelengths respectively.

#### 2.2.2. Synchronous fluorescence

Synchronous fluorescence spectra of Hb, both unbound and in complex with Nilvadipine, were recorded on a Jasco FP-750 spectrofluorometer (Tokyo, Japan), as described earlier.^17^ Hb was maintained at 5 μM, and Nilvadipine was added to achieve molar ratios of 1 : 0, 1 : 1, and 1 : 5 (Hb : Nilvadipine). For probing tyrosine residues, the excitation–emission wavelength offset (Δ*λ*) was set to 15 nm, while for tryptophan residues Δ*λ* was 60 nm. Emission scans were collected from 260 to 340 nm for tyrosine and from 280 to 400 nm for tryptophan.

#### 2.2.3. Soret band absorption spectroscopy

The Soret band absorption spectra of Hb, both in the absence and presence of Nilvadipine, were measured using a spectrophotometer (Thermo Scientific, USA). Hb (5 μM) was subjected to incubation with varying amounts of Nilvadipine in molar ratios of 1 : 0, 1 : 1, and 1 : 5. The absorption spectra of the incubated samples, as well as the blank samples (0.05 M phosphate buffer, pH 7.4), were then scanned in the 350–500 nm range at room temperature.

#### 2.2.4. 3D fluorescence spectroscopy

3D emission–excitation matrices for Hb were collected in the absence and presence of Nilvadipine using the Jasco FP-750 spectrofluorometer (Tokyo, Japan) under identical conditions to the steady-state and synchronous measurements.^18^ By scanning excitation wavelengths from 200 to 400 nm in 10 nm increments and recording emission spectra from 200 to 500 nm with the same step size, we generated 3D plots that reveal changes in peak positions and intensities across both axes. Two dominant fluorescence features were monitored: Peak 1, arising from tryptophan/tyrosine side chains, and Peak 2, corresponding to the peptide backbone.

#### 2.2.5. Far UV CD spectroscopy

Circular dichroism (CD) measurements were conducted employing the ChirascanPlus spectropolarimeter (Applied Photophysics, UK) equipped with a Peltier system. The CD spectra of native Hb (5 μM) and its complex with Nilvadipine (protein/ligand ratio 1 : 1 and 1 : 5) were recorded in the far-UV range spanning from 200 to 260 nm. In, order to correct for the baseline, sodium phosphate buffer (20 mM, pH 7.4) was utilized. Each spectrum shown represents the average of 3 scans.

#### 2.2.6. Molecular docking

To predict and characterize the binding mode of Nilvadipine to Hb, we performed molecular docking using the crystal structure of bovine hemoglobin (PDB ID: 2DN1) downloaded from the RCSB Protein Data Bank.^19^ The two-dimensional structure of Nilvadipine (CID: 4494) was retrieved from PubChem. Prior to docking, the Hb model was prepared in Discovery Studio Visualizer by deleting extraneous chains, heteroatoms, and crystallographic water molecules, then adding all hydrogen atoms to complete its valence states. Nilvadipine was energy-minimized using the Universal Force Field (UFF) to relieve any internal strain and optimize its geometry. Docking calculations were carried out with AutoDock Vina within the PyRx 0.8 environment as described previously.^20^ The Hb receptor was kept rigid, with all polar hydrogens and Gasteiger charges assigned, and saved as a PDBQT file. Nilvadipine was defined as fully flexible, with its torsional degrees of freedom determined automatically by AutoDockTools. We conducted a blind docking search over a grid box of 35 × 35 × 15 Å, centered on coordinates (36, 41, 37) Å, which yielded nine distinct binding poses. The lowest-energy conformation was selected for further analysis, and its key intermolecular contacts such as hydrogen bonds, hydrophobic interactions, and π-stacking were mapped using Discovery Studio Visualizer.^21^ Further, InstaDock was employed to validate the docking results and binding site.^22^ It consistency supports the reproducibility and robustness of our docking predictions.

#### 2.2.7. Molecular dynamics (MD) studies

Molecular dynamics (MD) simulations were carried out using GROMACS 2020.6 with the CHARMM36 all-atom force field, which has been widely validated for protein–ligand systems and heme-containing proteins such as hemoglobin, ensuring accurate representation of hydrogen bonding, hydrophobic interactions, and heme group stability. The topology of Nilvadipine was generated using the CHARMM General Force Field (CGenFF).^23^ The Hb–Nilvadipine complex was solvated in a cubic box with TIP3P water molecules, and charge neutrality was achieved by adding Na^+^/Cl^−^ counter-ions to mimic physiological ionic strength (0.15 M).^24^ Energy minimization was performed using the steepest descent algorithm, followed by a two-step equilibration process: 500 ps of NVT (constant number of particles, volume, and temperature) equilibration at 310 K using a velocity-rescaling thermostat, and 500 ps of NPT (constant number of particles, pressure, and temperature) equilibration at 1 bar using the Parrinello–Rahman barostat. The production run was conducted for 100 ns with a 2 fs integration timestep under periodic boundary conditions. The LINCS algorithm was used to constrain all bond lengths, and long-range electrostatic interactions were treated using the Particle Mesh Ewald (PME) method with a cutoff of 1.2 nm for short-range interactions. Coordinates were saved every 10 ps for trajectory analysis. Moreover, to ensure reproducibility, molecular dynamics simulations were performed in triplicate with different random velocity seeds. Post-simulation clustering was carried out with the GROMACS gmx cluster tool (RMSD cutoff 0.2 nm), and representative structures from the top three dominant and one minor clusters were analyzed.

## 3. Results

### 3.1. Characterization of Hb–nilvadipine interaction

**Fig. 1:**
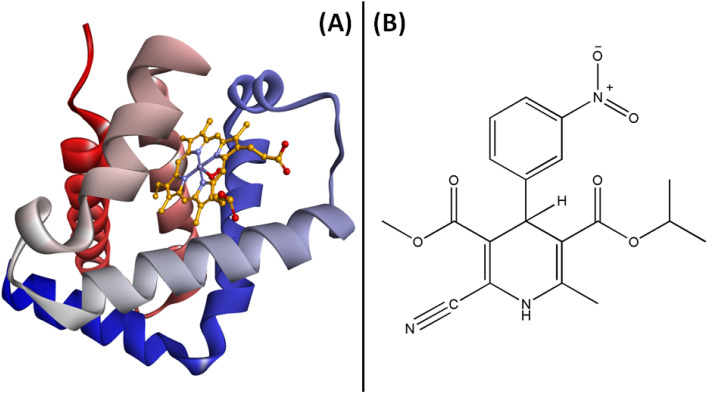
(A) Three-dimensional structure of Hb showing the relative positions of Trp19, and Trp61 residues, and (B) the two-dimensional structure of Nilvadipine.

Fluorescence quenching assays were utilized to probe the binding interaction between Hb and Nilvadipine. Intrinsic protein fluorophores such as tryptophan, tyrosine, and phenylalanine display environment-sensitive emission profiles, allowing us to monitor ligand-induced structural changes.^25^Fig. 1 shows the three-dimensional structure of Hb alongside the two-dimensional chemical structure of Nilvadipine.

**Fig. 2:**
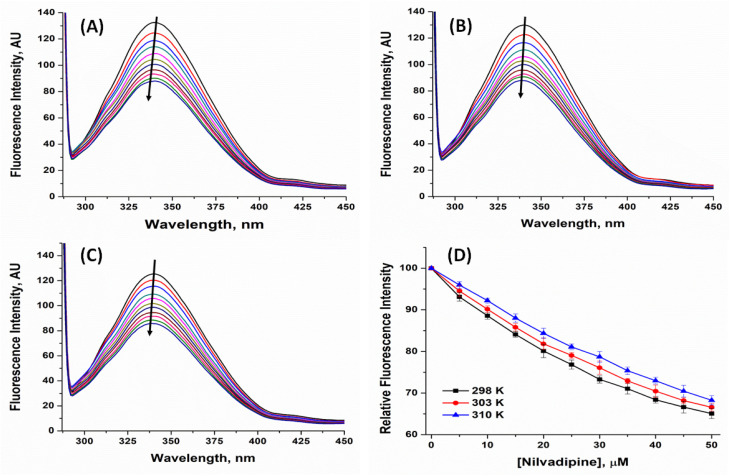
Fluorescence quenching in the intensity of Hb due to the binding of Nilvadipine at (A) 298 K, (B) 303 K, and (C) 310 K. The relative fluorescence intensity of Hb at different temperatures is shown in panel (D). Samples were excited at 295 nm, and emission was monitored from 300 to 450 nm with both excitation and emission bandwidths set to 5 nm. Hb was held constant at 5 μM in each measurement, while Nilvadipine concentrations were titrated from 0 to 50 μM in a total volume of 1.0 ml. All the experiments were conducted in triplicate.

We recorded fluorescence spectra of 5 μM Hb in the presence of Nilvadipine concentrations ranging from 0 to 50 μM at 298, 303, and 310 K (Fig. 2A–C). Increasing Nilvadipine concentration led to progressive quenching of Hb's emission and a 3 nm blue shift in the emission maximum (from 340 nm), indicating that tryptophan residues became more solvent-exposed or experienced a less hydrophobic microenvironment (Fig. 2D).

**Fig. 3:**
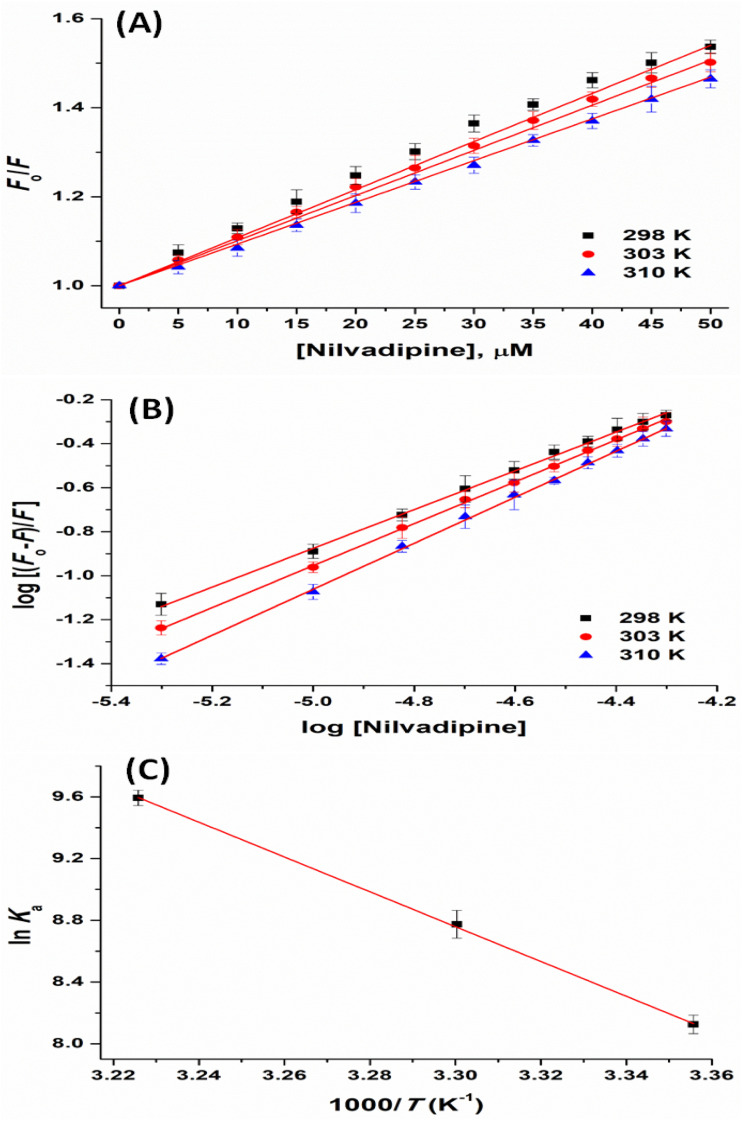
Binding and thermodynamics of Hb and Nilvadipine interaction. (A) Stern–Volmer plot, (B) Modified Stern–Volmer plot, and (C) van't Hoff plot. Samples were excited at 295 nm, and emission was monitored from 300 to 450 nm with both excitation and emission bandwidths set to 5 nm. Hb was held constant at 5 μM in each measurement, while Nilvadipine concentrations were titrated from 0 to 50 μM in a total volume of 1.0 ml. All the experiments were conducted in triplicate at 298, 303, and 310 K.

To quantify these effects, Stern–Volmer plots^26^ were constructed at 298, 303, and 310 K temperature (Fig. 3A) using eqn (2), yielding quenching constants (*K*_SV_) of 1.080 ± 0.012, 1.014 ± 0.009, and 0.937 ± 0.010 × 10^4^ M^−1^ respectively (Table 1).2
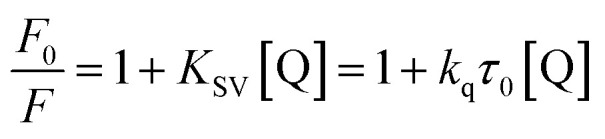
where, *F*_0_, *F*, *K*_SV_, *k*_q_, and *τ*_0_ are fluorescence intensity in the absence and presence of quencher (Q), Stern–Volmer quenching constant, bimolecular quenching constant, and lifetime of protein fluorescence in the absence of quencher (=5.71 × 10^−9^ s).^27^

**Table 1:** Binding parameters for the interaction of Nilvadipine with Hb

Temp. (K)	*K*_SV_ × 10^4^ (M^−1^)	*k*_q_ × 10^12^ (M^−1^ s^−1^)	*K*_a_ × 10^4^ (M^−1^)	*n*
298	1.080 ± 0.012	1.89 ± 0.06	0.3380 ± 0.003	0.8809 ± 0.04
303	1.014 ± 0.009	1.78 ± 0.07	0.6467 ± 0.005	0.9530 ± 0.07
310	0.937 ± 0.010	1.64 ± 0.04	1.4667 ± 0.006	1.0456 ± 0.07

The linearity of the Stern–Volmer relationship over the concentration range suggests either static or dynamic quenching. However, the calculated bimolecular quenching rate constants (*k*_q_) were approximately 10^12^ M^−1^ s^−1^ which was well above the diffusion-limited maximum of ∼10^10^ M^−1^ s^−1^, implying complex formation rather than collisional quenching. Furthermore, *K*_SV_ values decreased with increasing temperature, consistent with a static quenching mechanism in which higher thermal energy disrupts the noncovalent drug–protein complex. Together, these observations confirm that Nilvadipine binds to Hb to form a stable complex, rather than simply colliding with the protein in solution.

In our study, the conclusion of static quenching was based on the decreasing trend of Stern–Volmer constant (*K*_SV_) or increasing trend of binding affinity (*K*_a_) with increasing temperature. In addition, the magnitude of the quenching constants (*K*_q_) exceeds the diffusion-controlled limit of ∼10^10^ M^−1^ s^−1^. All of these observations are widely accepted indicators of complex formation in protein–ligand systems.^28,29^ These spectroscopic signatures collectively support the static quenching mechanism in the absence of fluorescence lifetime data. However, we acknowledge that fluorescence lifetime measurements remain the gold standard for confirming static quenching, as they can directly demonstrate whether excited-state decay kinetics are altered by complex formation. The absence of lifetime data in our study represents a limitation of the present study.

### 3.2. Determining binding and thermodynamic parameters of Hb–nilvadipine interaction

Binding and thermodynamic parameters for the Hb–Nilvadipine interaction were determined using a modified Stern–Volmer approach (eqn (3)).3
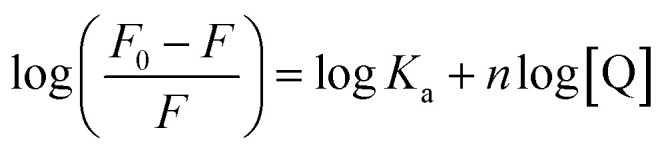
where, *K*_a_ is the binding constant.

Analysis of the resulting plot (Fig. 3B) and the data in Table 1 revealed that Nilvadipine binds to Hb with a 1 : 1 stoichiometry at 298 K, 303 K, and 310 K, as indicated by a binding-site number (*n*) close to unity (0.8809 to 1.0456 range). The association constants (*K*_a_) were 0.3380 ± 0.003, 0.6467 ± 0.005, and 1.4667 ± 0.006 × 10^4^ M^−1^ at 298, 303, and 310 K respectively. It is noteworthy that the value of *K*_a_ decreases with increasing temperature, indicating static quenching mechanism. Moreover, the values of *K*_a_ were in the range of 10^3^–10^5^ M^−1^, consistent with typical protein–ligand affinities reported.^30^

An estimation of Hb-bound fraction of Nilvadipine at therapeutic plasma concentrations was determined using the following relation:
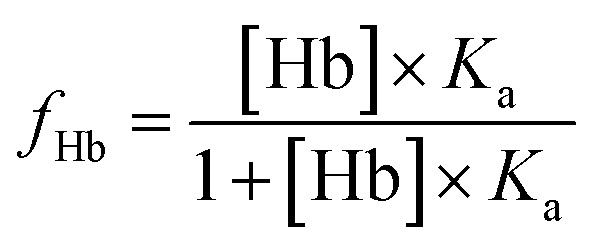
where, (*f*_Hb_) was Hb-bound fraction of the drug. [Hb] was taken as 2.3 mM (mean molar concentration of tetrameric Hb in whole blood) and *K*_a_ (=0.6467 × 10^4^ M^−1^ at 303 K) was derived from our experimental binding data. The *in vitro* binding constant predicts that approximately 93.7% of the free drug would associate with Hb. It indicates that Hb has a strong capacity to sequester Nilvadipine once the free drug is present in the erythrocyte environment. This high binding affinity suggests that, under assay conditions where the drug is freely available, most of it would be associated with Hb rather than remaining unbound in solution.

Since, enthalpy change (Δ*H*°) showed minimal variation over the temperature range studied, we applied standard van't Hoff and Gibbs–Helmholtz equations (eqn (4) and (5)) to extract Δ*H*° (change in enthalpy), Δ*S*° (change in entropy), and Δ*G*° (change in free-energy) values.4
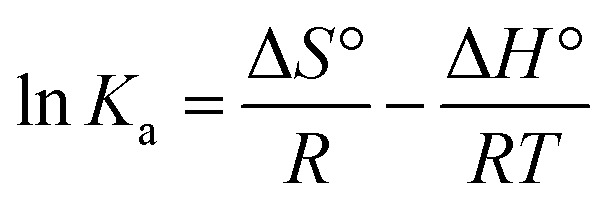

5 Δ*G*° = Δ*H*° − *T*Δ*S*° = −*RT* ln *K*_a_

where, *R* is the universal gas constant (=1.987 cal K^−1^ mol^−1^), and *T* is the temperature (Kelvin). The standard sign convention follows that a negative Δ*G*° value indicates a spontaneous binding process, while a negative Δ*H*° denotes an exothermic interaction, typically associated with hydrogen bonding and van der Waals forces. Similarly, a positive Δ*S*° reflects an increase in system disorder, often arising from hydrophobic effects or solvent release upon ligand binding.

In this study, plotting ln *K*_a_ against 1/*T* (Fig. 3C and Table 2) allowed the calculation of thermodynamic parameters. The positive Δ*H*° (=22.42 ± 0.17 kcal mol^−1^) and Δ*S*° (=91.39 ± 0.21 cal mol^−1^ K^−1^) values confirm that hydrophobic forces are the main drivers of complex formation, whereas the negative Δ*G*° (−4.81 ± 0.05, −5.24 ± 0.08, and −5.91 ± 0.06 kcal mol^−1^) at all three temperatures *i.e.* 298, 303, and 310 K respectively indicates a spontaneous binding process (Table 2).

**Table 2:** Thermodynamics parameters for the interaction of Nilvadipine with Hb

Temp. (K)	Δ*H*° (kcal mol^−1^)	Δ*S*° (cal mol^−1^ K^−1^)	*T*Δ*S*° (kcal mol^−1^)	Δ*G*° (kcal mol^−1^)
298	22.42 ± 0.17	91.39 ± 0.21	27.23 ± 0.17	−4.81 ± 0.05
303			27.69 ± 0.22	−5.27 ± 0.08
310			28.33 ± 0.19	−5.91 ± 0.06

It is worth noting that the Stern–Volmer quenching constant (*K*_SV_) and the association constant (*K*_a_) provide complementary insights. *K*_SV_ quantifies the efficiency of fluorescence quenching, and thus the proximity and dynamics of the quenching interaction. On the other hand, *K*_a_ measures the equilibrium affinity between Nilvadipine and Hb, reflecting the net result of specific intermolecular forces such as hydrophobic contacts, hydrogen bonding, and electrostatic interactions.

### 3.3. FRET between Hb and nilvadipine

Förster resonance energy transfer (FRET) is a spectroscopic approach for probing nanoscale distances and interactions between biomolecules. It capitalizes on non-radiative dipole–dipole coupling, whereby an excited donor fluorophore transfers energy to a proximal acceptor molecule.^31^ The efficiency of this process is highly sensitive to the separation between donor and acceptor: significant energy transfer only occurs when the donor's emission spectrum overlaps with the acceptor's absorption band. In our system, Hb serves as the fluorescent donor and Nilvadipine as the quencher (acceptor). By applying eqn (6)–(8), we calculated the overlap integral (*J*), the Förster distance (*R*_0_), the actual donor–acceptor separation (*r*), and the FRET efficiency (*E*).6
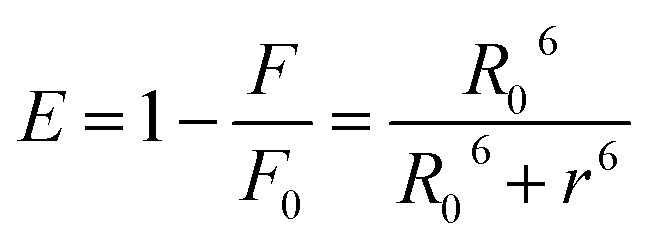
where *R*_0_ is the distance between the donor and acceptor at which energy transfer becomes 50%, and *r* is the actual distance between donor and acceptor molecules. *F*_0_ and *F* are the fluorescence intensities of the donor in the absence and presence of the acceptor, respectively.

*R*_0_ is determined as

7 *R*_0_^6^ = 8.79 × 10^−25^*K*^2^*n*^−4^*ϕJ*

where, *K*^2^ defines the geometry of the donor and acceptor dipoles (=2/3), *n* is the refractive index of the medium (=1.33), *ϕ* is the fluorescence quantum yield in the absence of the acceptor (=0.118), and *J* is the overlap integral of the donor's fluorescence spectra and the acceptor's absorption spectra.

*J* can be determined using the following relation,8
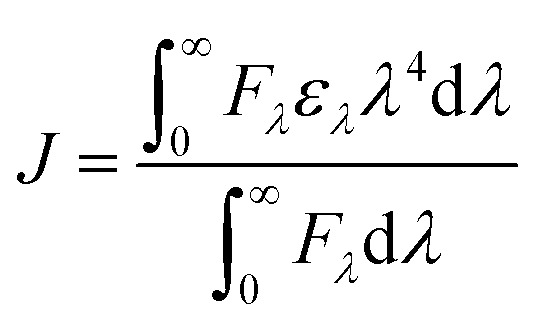
where, *F*_*λ*_ is the fluorescence intensity of the donor at wavelength *λ*, and *ε*_*λ*_ is the molar extinction coefficient of the acceptor at wavelength *λ*.

**Fig. 4:**
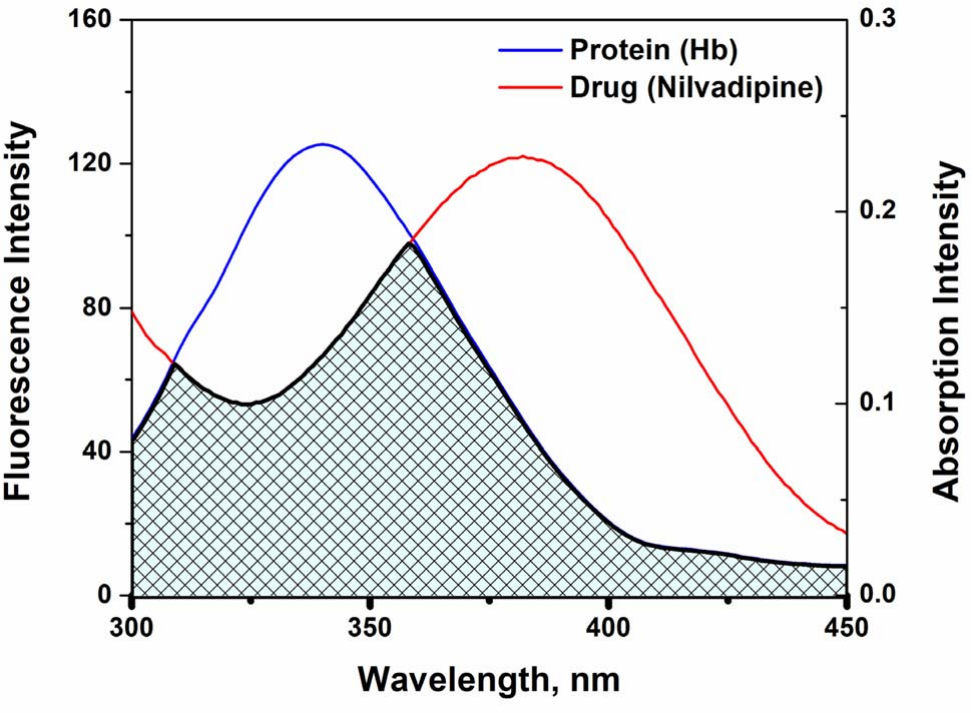
FRET between the normalized absorption spectrum of Nilvadipine, and the normalized fluorescence spectrum of Hb. The concentrations of Hb and Nilvadipine were set at 5 μM in 0.05 M phosphate buffer, pH 7.4 at 298 K.

As shown in Fig. 4 and Table 3, we obtained *J* = 1.854 × 10^−14^ M^−1^ cm^3^, *R*_0_ = 2.7253 nm, *r* = 3.0232 nm, and *E* = 34.49%. Both *R*_0_ and *r* lie within the 2–8 nm window that defines an effective FRET range, and the condition 0.5*R*_0_ ≤ *r* ≤ 1.5*R*_0_ confirms a static quenching mechanism arising from complex formation. Furthermore, as Nilvadipine's binding affinity (*K*_a_) for Hb increases, the average separation between donor and acceptor decreases, thereby enhancing FRET efficiency and reducing the apparent Förster distance (*r*), which is consistent with stronger molecular association.

**Table 3:** FRET parameters for the interaction between Hb and Nilvadipine

*J* (M^−1^ cm^3^)	*R*_0_ (nm)	*r* (nm)	*E* (%)
1.854 × 10^−14^	2.7253	3.0232	34.49

### 3.4. Analysis of molecular docking

Molecular docking enables detailed prediction of how small molecules fit within a protein's active or binding sites by exploring possible orientations and interactions at the atomic level.^32^ We extended our computational validation by performing blind docking using InstaDock.^22^ The outcomes consistently reproduced the same hemoglobin-binding pocket for Nilvadipine as identified in our original docking workflow (Fig. S1). This concordance across two independent docking platforms provides robust validation of the predicted binding site and enhances the methodological rigor and reproducibility of our findings. In our study, the top-ranked poses consistently converged on the same binding pocket involving residues ASN68, ASP64, LEU83, and LEU86 near the heme site. These residues overlap with literature-reported drug-binding regions in hemoglobin, supporting the validity and reproducibility of the docking results. Thus to characterize the Hb–Nilvadipine complex, out of the nine distinct docking poses, we selected the one with the lowest predicted binding energy as the most likely mode of interaction. As summarized in Table 4, the top-scoring model exhibited a binding energy of −5.50 kcal mol^−1^, indicating a moderately strong and thermodynamically favorable association. Notably, this value aligns closely with the −5.27 kcal mol^−1^ estimated from fluorescence quenching data at 303 K, underscoring the consistency between spectroscopic and computational findings.

**Table 4:** Molecular docking parameters for the interaction of Nilvadipine with Hb

Donor–acceptor pair	Distance (Å)	Nature of interaction	Docking energy (kcal mol^−1^)	Binding affinity (M^−1^)
ASN68:HD21 – LIG:N	2.49	Hydrogen bond	−5.5	1.08 × 10^4^
ASN68:HD22 – LIG:O	2.47	Hydrogen bond		
LIG:C – ASP64:O	3.57	Carbon hydrogen bond		
ALA82 – LIG:C	4.14	Hydrophobic (alkyl)		
LIG:C – LEU83	4.54	Hydrophobic (alkyl)		
LIG:C – LEU86	3.80	Hydrophobic (alkyl)		

**Fig. 5:**
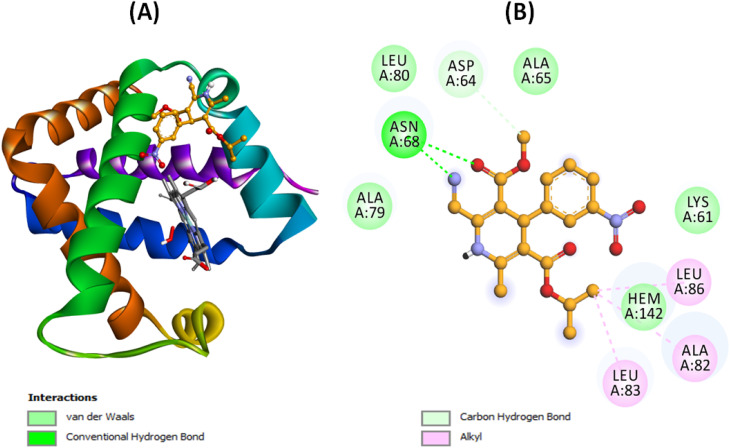
Molecular docking between Hb and Nilvadipine. (A) The best docked pose (with lowest energy) of Hb–Nilvadipine complex, and (B) Hb–Nilvadipine interaction plot indicating different kinds of interactions such as hydrogen bonds, hydrophobic (alkyl) and van der Waals interactions. AutoDock Vina enable in PyRx 0.8 environment was employed for conducting molecular docking.

Fig. 5A depicts the preferred docked conformation, highlighting key contacts between Nilvadipine and Hb residues. Two conventional hydrogen bonds are formed with the side-chain amide hydrogens of ASN68 (HD21 and HD22), and an additional carbonyl interaction involves ASP64:O. These specific interactions help orient the ligand within the binding pocket. Surrounding hydrophobic contacts namely alkyl interactions with ALA82, LEU83, and LEU86 along with van der Waals forces from residues such as LYS61, ALA65, ALA79, and LEU80, further stabilize the complex. Additionally, Nilvadipine makes a hydrophobic contact with the heme group, reinforcing its binding position (Fig. 5B). Together, these directional and nonspecific interactions underpin the structural integrity of the Hb–Nilvadipine complex. Several other drugs are known to bind hemoglobin at overlapping or distinct sites. For example, ibuprofen and other nonsteroidal anti-inflammatory drugs (NSAIDs) bind within the β-chain hydrophobic cavity, with PHE43, LEU83, and VAL67 again being critical contact points, indicating partial overlap with Nilvadipine's predicted site. In contrast, certain ligands such as quinine occupy alternative regions, with interaction hot spots including TYR42, LYS66, and ASP94, suggesting a different binding orientation and site compared to Nilvadipine. These variations underline that while Nilvadipine shares a binding locus with several hydrophobic-site ligands, there are also drugs that bind hemoglobin at distinct pockets, reflecting the structural versatility of hemoglobin in ligand accommodation.

### 3.5. Analysis of molecular dynamics (MD) simulation

**Fig. 6:**
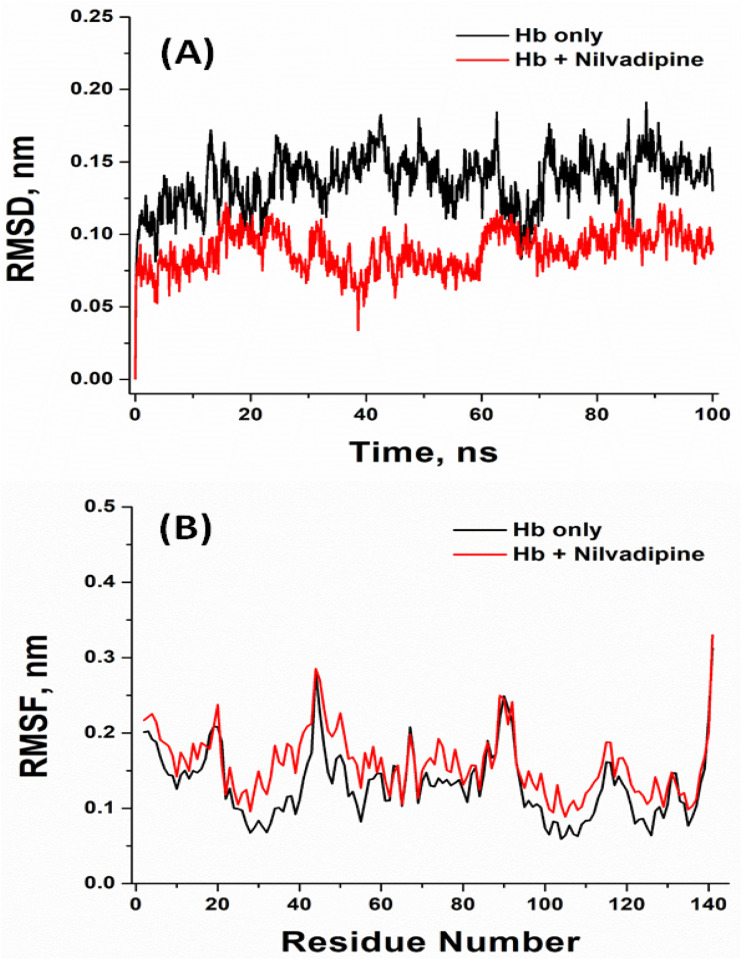
Molecular dynamics simulation of Hb–Nilvadipine complex. (A) Root mean square deviation (RMSD) plot and (B) root mean square fluctuation (RMSF) plot. The experiments were conducted in triplicate at 300 K using CHARMM36 force field in GROMACS 2020.6.

**Fig. 7:**
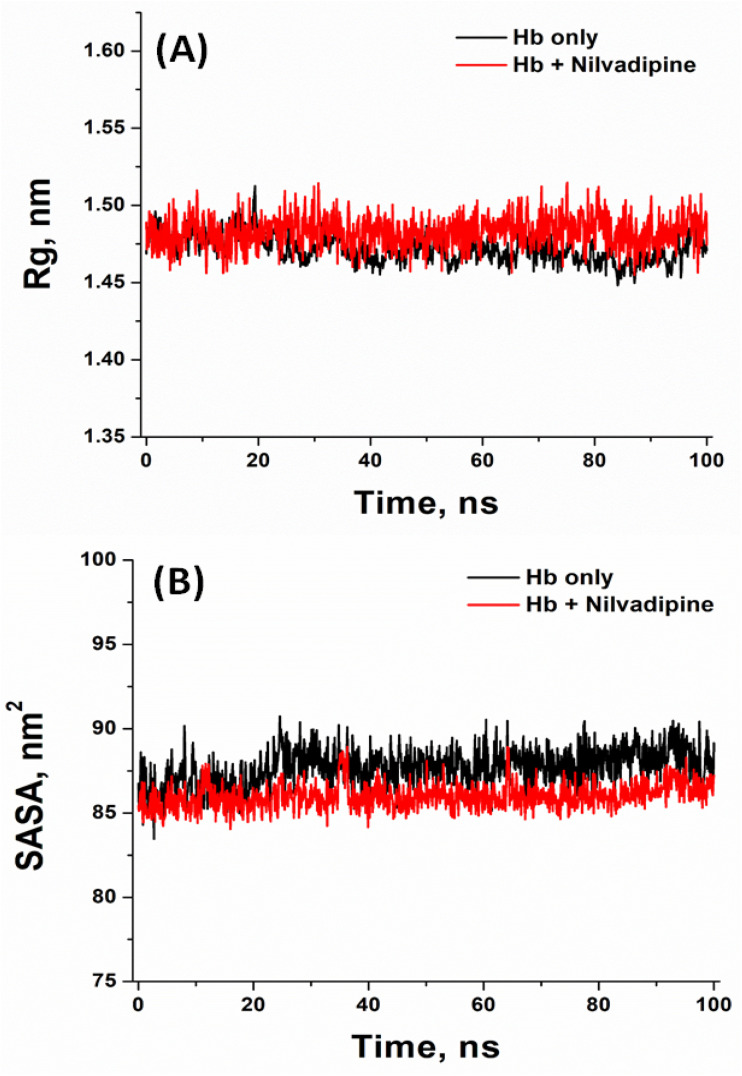
Molecular dynamics simulation of Hb–Nilvadipine complex. (A) Radius of gyration (*R*_g_) plot and (B) solvent accessible surface area (SASA). The experiments were conducted in triplicate at 300 K using CHARMM36 force field in GROMACS 2020.6.

Molecular dynamics (MD) simulations were carried out to examine both the intrinsic stability of unbound Hb and the behavior of the most favorable Hb–Nilvadipine complex over time. The 100 ns timescale was selected because previous studies have demonstrated that globular proteins such as hemoglobin achieve conformational equilibrium within this period, and our system exhibited stable root-mean-square deviation (RMSD) and fluctuation (RMSF), radius of gyration (*R*_g_), and solvent-accessible surface area (SASA) values well before the end of the trajectory. To ensure reproducibility, we performed three independent MD simulations using different random seeds. The simulations showed consistent convergence across key structural parameters, including RMSD, RMSF, *R*_g_, and SASA (Fig. S2–S5). The results presented in Fig. 6 and 7 represent the mean values obtained from these three independent runs.

We monitored the RMSD of backbone atoms to gauge how much the protein deviated from its starting conformation throughout the 100 ns trajectories. As shown in Fig. 6A, free Hb (black trace) remained largely stable after an initial equilibration, fluctuating between 0.080 and 0.191 nm (mean ± SD = 0.139 ± 0.021 nm) from 20 to 100 ns. The Hb–Nilvadipine complex settled more quickly, after roughly 20 ns, and maintained deviations of 0.034–0.124 nm (mean ± SD = 0.089 ± 0.027 nm). Since both systems stayed well under the 0.20 nm threshold commonly used to define structural stability, these results indicate that Nilvadipine binding does not destabilize the overall protein fold.

To pinpoint regions of varying flexibility, per-residue RMSF values were calculated (Fig. 6B). The fluctuation profiles of free and ligand-bound Hb were largely similar, with no residue showing mobility greater than 0.3 nm. Notably, residues 20–60 exhibited slightly higher RMSF in the complex, suggesting localized increases in flexibility upon Nilvadipine binding; however, the absence of large outliers confirms that both the apo form and the complex maintain a coherent structural ensemble.

We used the radius of gyration (*R*_g_) to assess overall compactness of Hb in the absence and presence of Nilvadipine (Fig. 7A). The minimum and maximum *R*_g_ of free Hb during 20–100 ns MD simulation were 1.45 nm, and 1.51 nm, while *R*_g_ of Hb–Nilvadipine complex fluctuated in 1.38–1.60 nm range. Free Hb averaged an *R*_g_ of 1.472 ± 0.009 nm, whereas the Hb–Nilvadipine complex was marginally less compact (1.484 ± 0.010 nm). This subtle reduction in *R*_g_ upon ligand binding implies a slight bulging of the protein's tertiary structure.

Finally, we tracked the SASA to measure how exposure to solvent changes with ligand binding (Fig. 7B). Unbound Hb showed SASA values ranging from 83.46 to 90.73 nm^2^ (mean = 87.71 ± 6.05 nm^2^), while the complex ranged from 84.05 to 88.93 nm^2^ (mean = 86.01 ± 5.32 nm^2^). The comparable SASA profiles indicate that Nilvadipine remains snugly anchored within its binding sites without significantly altering the protein's overall solvent exposure, supporting the notion of a stable protein–ligand assembly.

Further, to evaluate the reproducibility and stability of the Hb–Nilvadipine interaction, post-simulation clustering was performed on the 100 ns MD trajectory using GROMACS gmx cluster tool with RMSD cutoff of 0.2 nm. The trajectory was partitioned into three dominant clusters and a minor cluster (Fig. S6), with Cluster 1 accounting for ∼52% of the total conformations, Cluster 2 for ∼31%, and Cluster 3 for ∼12%, while minor cluster 4 contributed less than 5% (Table S1). In Cluster 1, Nilvadipine was deeply embedded within the hydrophobic pocket formed by residues ASP64, ASN68, and LEU83 with stable hydrogen bonding to ASP64, ASN68 and Heme along with hydrophobic packing against the Heme group and LYS61, consistent with the docking-predicted binding pose. Cluster 2 revealed a tilted orientation stabilized by hydrogen bond with ASN68, and hydrophobic interactions with ALA82, LEU83, and Heme. Similarly, Cluster 3 presented a shallower orientation at the pocket entrance with partial solvent exposure, supported by transient hydrogen bonding with LYS61, and ASN68 along with hydrophobic interactions with LEU83, and Heme. Importantly, the cluster population analysis showed that Nilvadipine predominantly adopts a stable, hydrophobically driven binding mode, reinforcing the reliability of the docking results. These findings highlight that despite local conformational variations, the Hb binding site maintains a consistent drug-binding environment, supporting the biological relevance of the Hb–Nilvadipine interaction. Furthermore, the trajectory overlays of key Hb residues (ASP64, ASN68, ALA82, LEU83, and LEU86) clearly show that these residues remain stably positioned around the ligand throughout the 100 ns simulation, with reduced fluctuations (<0.1 nm) (Fig. S7).

### 3.6. Conformational changes in Hb due to interaction with nilvadipine

#### 3.6.1. Soret band absorption spectroscopy

**Fig. 8:**
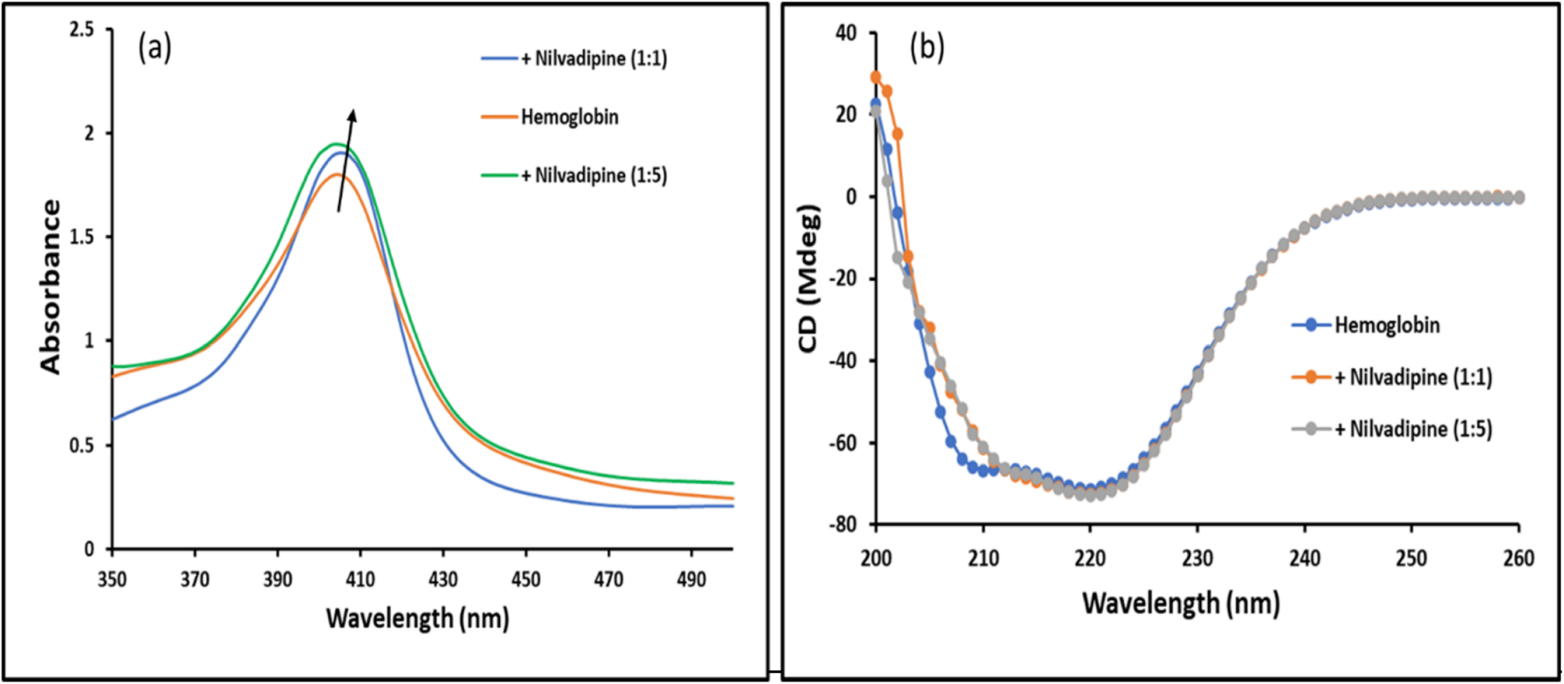
Conformational change in Hb due to the binding of Nilvadipine. (a) Soret band absorption spectra and (b) Far-UV CD spectra. Hb (5 μM) was subjected to incubation with varying amounts of Nilvadipine in molar ratios of 1 : 0, 1 : 1, and 1 : 5. The experiments were conducted in 0.05 M phosphate buffer, pH 7.4 at 298 K.

Soret band absorption spectroscopy is a widely employed technique for evaluating the conformational changes resulting from the protein–drug interaction. The spectra of Hb in the absence of Nilvadipine displayed a characteristic peak at approximately 410 nm wavelength, indicating the compactness of the three-dimensional structure around heme groups, as depicted in Fig. 8a. Upon the subsequent addition of Nilvadipine, the intensity at heme absorbing groups slightly increased with a minor red shift in wavelength maxima (Fig. 8a). These observations confirm that the interaction of Nilvadipine with Hb induces a slight alteration in its three-dimensional structure.

#### 3.6.2. Far-UV CD analysis

CD spectroscopy was employed in this study to elucidate the conformations of the protein in the presence or absence of a compound under various experimental conditions. Far-UV CD spectra of Hb were recorded in the presence or absence of varying concentrations of Nilvadipine, as depicted in Fig. 8b. The findings revealed that Hb in the absence of Nilvadipine exhibited a characteristic CD spectrum typical of α-helix protein. However, in the presence of Nilvadipine at 1 : 1 and 1 : 5 molar ratios, CD spectrum was insignificantly altered (Fig. 8b).

#### 3.6.3. Synchronous fluorescence analysis

**Fig. 9:**
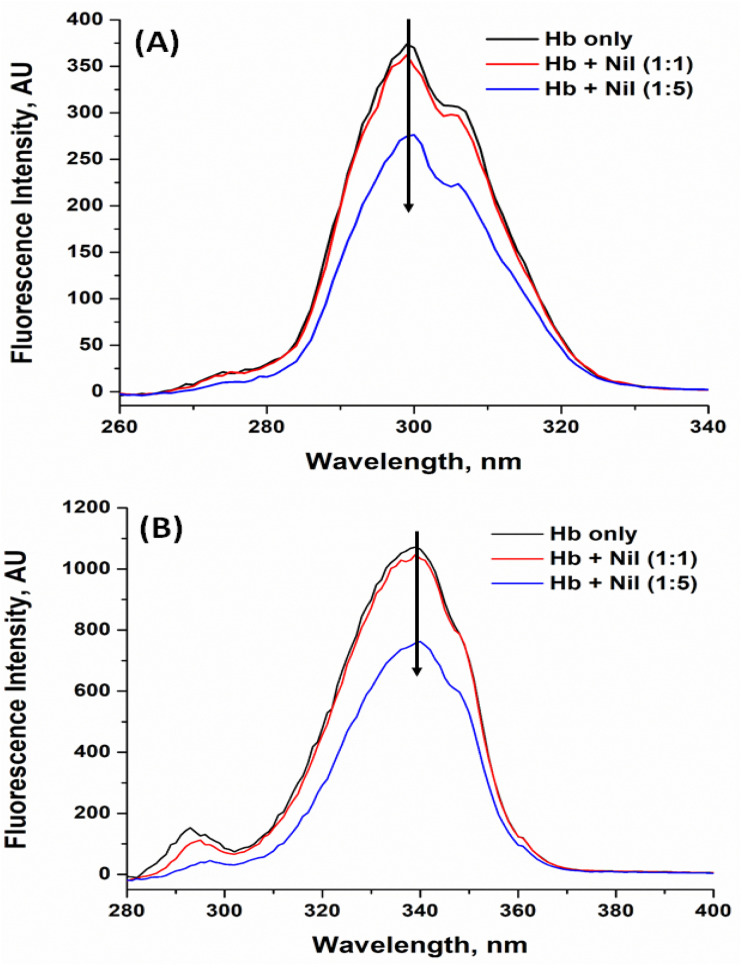
Synchronous fluorescence of Hb in the presence of Nilvadipine. (A) Δ15 (for microenvironment of Tyr residues), and (B) Δ60 (for microenvironment of Trp residues). Concentration of Hb was set at 5 μM, and the concentration of Nilvadipine was varied in 0–25 μM range in 1 : 0, 1 : 1 and 1 : 5 molar ratio. Emission scans for tyrosine and tryptophan were collected from 260–340 nm and 280–400 nm range respectively. The experiments were conducted in 0.05 M phosphate buffer, pH 7.4 at 298 K.

We employed synchronous fluorescence spectroscopy to monitor how Nilvadipine binding alters the local environments of Hb's aromatic residues. By scanning excitation and emission wavelengths simultaneously with offsets (Δ*λ*) of 15 nm and 60 nm, we selectively probed tyrosine and tryptophan microenvironments, respectively. As Nilvadipine concentration increased (Hb : Nilvadipine molar ratios of 1 : 0, 1 : 1, and 1 : 5), both Δ*λ*15 and Δ*λ*60 spectra showed progressive decreases in fluorescence intensity (Fig. 9). This quenching, together with slight shifts in peak positions, indicates that binding increases local polarity and disrupts hydrophobic contacts around these residues, consistent with a ligand-induced rearrangement of their surroundings.

#### 3.6.4. 3D fluorescence spectral analysis

**Fig. 10:**
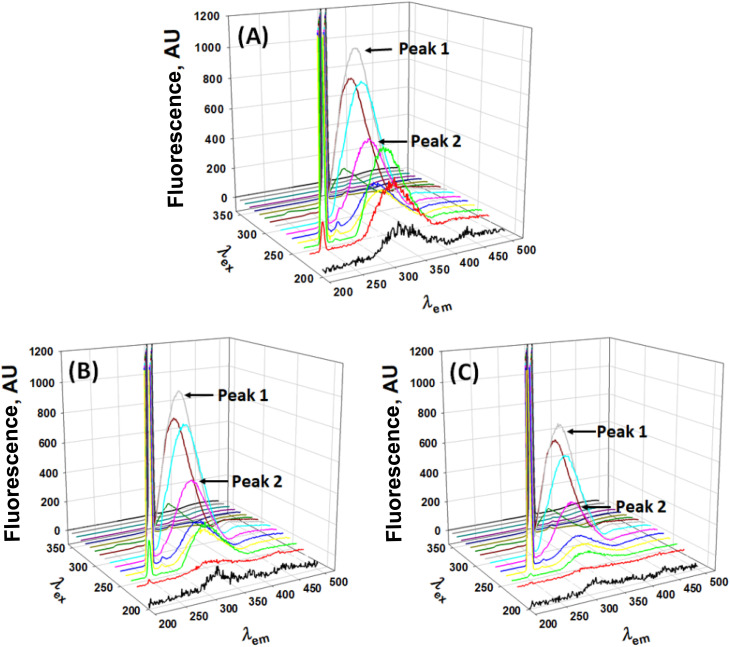
3D fluorescence spectra of Hb at different Hb : Nilvadipine molar ratios. (A) 1 : 0, (B) 1 : 1, and (C) 1 : 5. Concentration of Hb was set at 5 μM, and the concentration of Nilvadipine was varied in 0–25 μM range in 1 : 0, 1 : 1 and 1 : 5 molar ratio. The excitation wavelengths were scanned from 200 to 400 nm in 10 nm increments and emission spectra were recorded from 200 to 500 nm with the same step size (10 nm). Peak 1 indicated the fluorescence signal arising from tryptophan/tyrosine side chains, while Peak 2 corresponded to the peptide backbone. The experiments were conducted in 0.05 M phosphate buffer, pH 7.4 at 298 K.

3D fluorescence mapping further elucidated conformational changes across the protein. The 3D emission–excitation surfaces for free Hb and its complexes with Nilvadipine (1 : 1 and 1 : 5 ratios) are presented in Fig. 10. Two principal peaks were analyzed: Peak 1 corresponds to aromatic side chains (Trp/Tyr), and Peak 2 reflects the peptide backbone. At a 1 : 1 ratio, Peak 1 intensity dropped by 5% (with a 2 nm blue shift) while Peak 2 decreased by 49.1% (Table 5). At 1 : 5 ratio, reductions reached 24.4% for Peak 1 (3 nm blue shift) and 85.8% for Peak 2. These changes imply that tryptophan residues become less solvent-exposed and that the overall secondary structure of Hb is partially disrupted upon Nilvadipine association.

**Table 5:** 3D-fluorescence parameters for the interaction between Hb and Nilvadipine

Condition	Peak no.	Peak position [*λ*_ex_/*λ*_em_ (nm nm^−1^)]	Peak intensity	Percentage (%)
Hb only	1	280/334	1046	100
	2	230/326	523	100
Hb + nilvadipine (1 : 1)	1	280/332	994	95.0
	2	230/326	266	50.9
Hb + nilvadipine (1 : 5)	1	280/331	791	75.6
	2	230/326	74	14.2

Combined with circular dichroism, intrinsic fluorescence, and Soret band absorbance data, these synchronous and 3D fluorescence results demonstrated that Nilvadipine binding induces subtle but measurable conformational alterations in Hb. Integrating spectroscopic observations with computational docking and dynamics provided a comprehensive view of the Hb–Nilvadipine complex, shedding light on its structural and functional implications for drug delivery and protein-based biotechnologies.

## 4. Discussion

In this study, we probed the interaction of an anti-hypertensive drug (Nilvadipine, dihydropyridine calcium channel blocker) with hemoglobin (Hb) using various spectroscopic and computational approaches.

### 4.1. Binding of nilvadipine to Hb through fluorescence quenching

Our spectroscopic investigations revealed that Nilvadipine induces significant static fluorescence quenching of hemoglobin (Hb), with Stern–Volmer constants (*K*_SV_) on the order of 10^4^ M^−1^ and association constants (*K*_a_) ranging from 3.380 × 10^3^ to 1.4667 × 10^4^ M^−1^, which declined with increasing temperature. Comparative assessment with other dihydropyridine calcium channel blockers highlights both similarities in their Hb-binding characteristics. Nilvadipine exhibited a moderate binding affinity, comparable to levamlodipine (∼1 × 10^4^ M^−1^).^28^ Such comparisons underscore that Hb-binding is not merely an *in vitro* artifact but a potential determinant of pharmacokinetic behavior (Table 6).

**Table 6:** Comparative analysis of dihydropyridine drug–hemoglobin interactions

Drug	Binding constant (*K*_a_)	Key interacting residues	Thermodynamic parameters	Binding mechanism	Pharmacokinetic implications	References
Nilvadipine	∼0.338–1.4667 × 10^4^ M^−1^	ASN68, ASP64 (H-bonds); ALA82, LEU83, LEU86 (hydrophobic, alkyl contacts); heme interaction	Δ*H*° > 0, Δ*S*° > 0 (hydrophobic forces dominate); Δ*G*° < 0 (spontaneous)	Static quenching, stable 1 : 1 Hb–drug complex	Hb may act as a transient reservoir, potentially modulating free fraction and volume of distribution	Present study
Levamlodipine	∼1 × 10^4^ M^−1^	TYR35 and ASP108 residues form H-bonds; partial overlap with heme pocket	Δ*H*° < 0, Δ*S*° < 0 (H-bonding and van der Waals' interactions are dominant); Δ*G*° < 0	Static quenching	Stronger Hb association than nilvadipine, possibly reducing free drug fraction and prolonging circulation	28

From a pharmacokinetic standpoint, these molecular differences likely influence drug distribution and systemic exposure. Nilvadipine's moderate Hb affinity suggests that erythrocytes may act as a temporary reservoir, sequestering drug without dramatically lowering the free plasma fraction. In contrast, levamlodipine's stronger Hb association may prolong circulation time but reduce immediate availability, consistent with its long half-life. These mechanistic insights align with *in vivo* pharmacokinetic data, where levamlodipine is characterized by high bioavailability and a prolonged half-life.^28^

Estimation of the binding constant further predicts that ∼93.7% of the free Nilvadipine in the erythrocyte environment would associate with Hb. While this indicates strong sequestration *in vitro*, the *in vivo* relevance is moderated by the fact that Nilvadipine is already >97% bound to plasma proteins, mainly albumin and lipoproteins.^33^ Thus, only a small fraction of the total circulating drug is free to enter erythrocytes and interact with Hb, meaning the systemic contribution of Hb binding is secondary to plasma protein binding. Nevertheless, Hb binding could still serve as an ancillary reservoir, buffering drug levels and influencing whole blood *versus* plasma drug concentrations. Clinical factors such as anemia, hemolysis, or polycythemia may alter this balance, thereby modulating the pharmacological impact of Hb binding in specific patient populations.

### 4.2. Thermodynamics of Hb–nilvadipine interaction

Molecular-level insights reveal that Nilvadipine associates with Hb primarily through hydrophobic stabilization within a defined pocket, reinforced by hydrogen bonds with ASN68 and ASP64 and alkyl contacts with residues such as ALA82, LEU83, and LEU86, along with favorable interactions with the heme moiety. Thermodynamic analysis further supports this mechanism, as positive Δ*H*° and Δ*S*° values indicate a hydrophobic, entropy-driven interaction, while negative Δ*G*° confirms spontaneity. These thermodynamic signatures are consistent with reported dihydropyridine–protein interactions, where hydrophobic contacts dominate. For instance, Wani *et al.* demonstrated similar positive enthalpy and entropy changes across several protein–drug systems, attributing binding to the release of structured water molecules from hydrophobic surfaces.^34^ Likewise, clonazepam–tau protein binding exhibited comparable profiles, with static quenching, positive Δ*S*°, and hydrophobic interactions inferred from temperature-dependent *K*_SV_ trends.^35^ In contrast, other dihydropyridines show distinct binding modes: levamlodipine primarily interacts *via* hydrogen bonding and van der Waals interactions (Δ*H*° < 0, Δ*S*° < 0) (Table 6). These distinctions suggest that while Nilvadipine, and levamlodipine form stable Hb complexes, the underlying physicochemical forces differ, potentially influencing their sensitivity to physiological variables such as pH, temperature, and the presence of competing ligands.

### 4.3. Binding of nilvadipine to Hb through molecular docking

Computational docking produced a binding energy of −5.50 kcal mol^−1^, matching the −5.27 kcal mol^−1^ derived from quenching data. Our docking-predicted binding energy is in close agreement with the thermodynamically derived value, mirroring trends seen in other drug–Hb studies. For example, levamlodipine docking reported binding energies around −5.4 kcal mol^−1^, with similar hydrogen-bond and hydrophobic networks stabilizing the complex.^28^ More generally, structure-based docking across diverse targets frequently yields binding energies in the −5 to −8 kcal mol^−1^ range for moderate-affinity ligands.^36,37^ The concurrence of our computational and experimental energies strengthens confidence in the proposed binding mode.

### 4.4. Molecular dynamics (MD) simulation analysis of nilvadipine to Hb interaction

Molecular dynamics (MD) simulation results provided insights into the structural and functional implications of Nilvadipine binding to Hb. The RMSD analysis revealed that the Hb–Nilvadipine complex remained highly stable throughout the trajectory, with fluctuations consistently below 0.20 nm. Such low RMSD values indicate that Nilvadipine binding does not perturb the overall structural framework of Hb, suggesting that the protein preserves its native conformation required for physiological activity. Similar results are reported in previous study wherein MD studies of levamlodipine–Hb complexes documented equilibrium RMSD values under 0.20 nm and stable hydrogen-bonding networks throughout 100 ns simulations.^28^ The RMSF analysis showed limited residue-level flexibility (<0.30 nm for most residues), with only minor increases around loop regions. Importantly, residues near the heme pocket and key secondary structures (α-helices) exhibited minimal fluctuations, confirming that the structural integrity of Hb's oxygen-binding regions remained intact. These findings imply that Nilvadipine binding does not interfere with the dynamic motions required for cooperative oxygen binding and release. Moreover, *R*_g_ of the Hb–Nilvadipine complex demonstrated a slight increase compared with free Hb, indicative of marginal relaxation of the globin fold. However, this relaxation was within physiologically acceptable limits and did not distort the heme-binding pocket or alter subunit packing, both of which are critical for Hb's oxygen transport function. Similarly, SASA analysis revealed minimal changes upon ligand binding, suggesting that Nilvadipine does not significantly alter solvent exposure of functional residues or disrupt Hb's interactions with physiological ligands such as oxygen, and carbon dioxide. Instead, Nilvadipine appears to occupy a hydrophobic binding pocket without hindering Hb's allosteric regulation or gas exchange capacity. The limited SASA changes in our study further confirm that Nilvadipine remains buried within Hb's hydrophobic pockets, a pattern echoed in both spectroscopic–computational investigations.^34^ From a functional perspective, these findings suggest that Nilvadipine's association with Hb does not compromise its primary physiological role in oxygen transport. Instead, Hb may act as a transient reservoir that sequesters the drug without impairing ligand accessibility to heme iron. This stabilization effect aligns with our thermodynamic data, which indicated hydrophobic and spontaneous binding. Taken together, the MD simulations not only confirm the stable binding of Nilvadipine to Hb but also provide mechanistic insight into how this interaction may influence drug distribution in circulation without adversely affecting Hb's essential biological function. Similar pharmacokinetic effects have been described for other dihydropyridines, where Hb interactions modulate free-drug equilibrium and tissue uptake.^28^

## 5. Limitations of the study

This study is specifically focused on the characterization of Nilvadipine–Hb interactions, and while the results provide valuable mechanistic insights, broader generalizations to other drug–Hb systems would require validation with additional compounds and statistical comparisons. The spectroscopic signatures obtained consistently support a static quenching mechanism, even though fluorescence lifetime data are not included. We acknowledge that fluorescence lifetime analysis is the gold standard for confirming static quenching, as it directly probes changes in excited-state decay kinetics. However, due to instrumental constraints, such measurements could not be performed in the present study. Nevertheless, the combination of temperature-dependent quenching constants, thermodynamic parameters, and docking analyses provides converging evidence for static quenching and a hydrophobic-driven binding mechanism. The absence of lifetime data is thus recognized as a limitation but does not undermine the reliability of the mechanistic conclusions drawn in this study.

## 6. Conclusion

Overall, this study provides a comprehensive molecular-level understanding of Hb–Nilvadipine interactions, revealing that the drug forms a stable, hydrophobically driven 1 : 1 complex within a defined binding pocket without altering Hb's structural integrity. The integration of spectroscopic, thermodynamic, docking, and molecular dynamics approaches not only validates the binding mode but also demonstrates Hb's role as a potential transient reservoir that may modulate Nilvadipine's pharmacokinetic behavior. These findings underscore the utility of Hb as a versatile model for probing drug–protein interactions and offer valuable insights that can inform rational drug design, delivery strategies, and safety evaluation for pharmacologically active small molecules.

## Conflicts of interest

The authors declare that they have no known competing financial interests or personal relationships that could have appeared to influence the work reported in this paper.

## Supplementary Material

RA-015-D5RA04162G-s001

## Data Availability

The data supporting this study are available upon reasonable request from the corresponding author (moskhan@ksu.edu.sa). Supplementary information is available. See DOI: https://doi.org/10.1039/d5ra04162g.

## References

[cit1] Rehman M. T., Khan A. U. (2015). Understanding the Interaction Between Human Serum Albumin and Anti-Bacterial/Anti-Cancer Compounds. Curr. Pharm. Des..

[cit2] Ali M. S., Rehman M. T., Al-Lohedan H. A., AlAjmi M. F. (2022). Exploration of the binding between cuminol and bovine serum albumin through spectroscopic, molecular docking and molecular dynamics methods. J. Biomol. Struct. Dyn..

[cit3] Otto C. N. , Hemoglobin metabolism, Rodak's Hematol. Clin. Princ. Appl., 2020, pp. 91–103

[cit4] Perutz M. F. (1970). Stereochemistry of cooperative effects in haemoglobin: Haem-Haem interaction and the problem of allostery. Nature.

[cit5] Ascenzi P., Bocedi A., Visca P., Altruda F., Tolosano E., Beringhelli T., Fasano M. (2005). Hemoglobin and heme scavenging. IUBMB Life.

[cit6] Seal P., Sikdar J., Roy A., Haldar R. (2018). Binding of ibuprofen to human hemoglobin: elucidation of their molecular recognition by spectroscopy, calorimetry, and molecular modeling techniques. J. Biomol. Struct. Dyn..

[cit7] Dohare N., Siddiquee M. A., Parray M. d., Kumar A., Patel R. (2020). Esterase activity and interaction of human hemoglobin with diclofenac sodium: A spectroscopic and molecular docking study. J. Mol. Recognit..

[cit8] Fang Q., Xing M., Guo C., Liu Y. (2017). Probing the interaction of doxycycline to trypsin and bovine hemoglobin by using multi-spectral techniques and molecular docking. J. Mol. Liq..

[cit9] Khan S. N., Islam B., Yennamalli R., Zia Q., Subbarao N., Khan A. U. (2008). Characterization of doxorubicin binding site and drug induced alteration in the functionally important structural state of oxyhemoglobin. J. Pharm. Biomed. Anal..

[cit10] Takabatake T., Yamamoto Y., Nakamura S., Hashimoto N., Satoh S., Yamada Y., Ohta H., Hattori N. (1987). Effect of the calcium antagonist Nilvadipine on haemodynamics at rest and during cold stimulation in essential hypertension. Eur. J. Clin. Pharmacol..

[cit11] Morin A., Mouzon B., Ferguson S., Paris D., Browning M., Stewart W., Mullan M., Crawford F. (2020). Nilvadipine suppresses inflammation via inhibition of P-SYK and restores spatial memory deficits in a mouse model of repetitive mild TBI. Acta Neuropathol. Commun..

[cit12] Lawlor B., Segurado R., Kennelly S., Olde Rikkert M. G. M., Howard R., Pasquier F., Börjesson-Hanson A., Tsolaki M., Lucca U., Molloy D. W. (2018). *et al.*, Nilvadipine in mild to moderate Alzheimer disease: A randomised controlled trial. PLoS Med..

[cit13] Paris D., Bachmeier C., Patel N., Quadros A., Volmar C.-H., Laporte V., Ganey J., Beaulieu-Abdelahad D., Ait-Ghezala G., Crawford F. (2011). *et al.*, Selective Antihypertensive Dihydropyridines Lower Aβ Accumulation by Targeting both the Production and the Clearance of Aβ across the Blood–Brain Barrier. Mol. Med..

[cit14] de Jong D. L. K., de Heus R. A. A., Rijpma A., Donders R., Olde Rikkert M. G. M., Günther M., Lawlor B. A., van Osch M. J. P., Claassen J. A. H. R. (2019). Effects of Nilvadipine on Cerebral Blood Flow in Patients With Alzheimer Disease. Hypertension.

[cit15] AlAjmi M. F., Rehman M. T., Khan R. A., Khan M. A., Muteeb G., Khan M. S., Noman O. M., Alsalme A., Hussain A. (2020). Understanding the interaction between α-1-acid glycoprotein (AGP) and potential
Cu/Zn metallo-drugs of benzimidazole derived organic motifs: A multi-spectroscopic and molecular docking study. Spectrochim. Acta, Part A.

[cit16] Weitner T., Friganović T., Šakić D. (2022). Inner Filter Effect Correction for Fluorescence Measurements in Microplates Using Variable Vertical Axis Focus. Anal. Chem..

[cit17] Rehman M. T., Shamsi H., Khan A. U. (2014). Insight into the binding mechanism of imipenem to human serum albumin by spectroscopic and computational approaches. Mol. Pharm..

[cit18] Rahman S., Rehman M. T., Rabbani G., Khan P., Alajmi M. F., Hassan M. I., Muteeb G., Kim J. (2019). Insight of the interaction between 2,4-thiazolidinedione and human serum albumin: A spectroscopic, thermodynamic and molecular docking study. Int. J. Mol. Sci..

[cit19] Park S.-Y., Yokoyama T., Shibayama N., Shiro Y., Tame J. R. H. (2006). 1.25 Å Resolution Crystal Structures of Human Haemoglobin in the Oxy, Deoxy and Carbonmonoxy Forms. J. Mol. Biol..

[cit20] Jabir N. R., Shakil S., Tabrez S., Khan M. S., Rehman M. T., Ahmed B. A. (2021). In silico screening of glycogen synthase kinase-3β targeted ligands against acetylcholinesterase and its probable relevance to Alzheimer's disease. J. Biomol. Struct. Dyn..

[cit21] San Diego: Accelrys Software Inc. , Discovery Studio Modeling Environment, Release 3.5

[cit22] Mohammad T., Mathur Y., Hassan M. I. (2021). InstaDock: A single-click graphical user interface for molecular docking-based virtual high-throughput screening. Briefings Bioinf..

[cit23] Huang J., Rauscher S., Nawrocki G., Ran T., Feig M., de Groot B. L., Grubmüller H., MacKerell A. D. (2017). CHARMM36m: an improved force field for folded and intrinsically disordered proteins. Nat. Methods.

[cit24] Price D. J., Brooks C. L. (2004). A modified TIP3P water potential for simulation with Ewald summation. J. Chem. Phys..

[cit25] Alqahtani A. S., Hidayathulla S., Rehman M. T., Elgamal A. A., Al-Massarani S., Razmovski-Naumovski V., Alqahtani M. S., El Dib R. A., Alajmi M. F. (2019). Alpha-amylase and alpha-glucosidase enzyme inhibition and antioxidant potential of 3-oxolupenal and katononic acid isolated from Nuxia oppositifolia. Biomolecules.

[cit26] Stern O., Volmer M. (1919). über Die Abklingzeit der Fluoreszenz. Z. Phys..

[cit27] Ware W. R. (1962). Oxygen Quenching Of Fluorescence In Solution: An Experimental Study Of The Diffusion Process. J. Phys. Chem..

[cit28] Xu L., Liu Z., Liao T., Tuo X. (2019). Probing the interaction between levamlodipine and hemoglobin based on spectroscopic and molecular docking methods. Spectrochim. Acta, Part A.

[cit29] Terenteva E. A., Apyari V. V., Dmitrienko S. G., Zolotov Y. A. (2015). Formation of plasmonic silver nanoparticles by flavonoid reduction: A comparative study and application for determination of these substances. Spectrochim. Acta, Part A.

[cit30] Aneja B., Khan N. S., Khan P., Queen A., Hussain A., Rehman M. T., Alajmi M. F., El-Seedi H. R., Ali S., Hassan M. I. (2019). *et al.*, Design and development of Isatin-triazole hydrazones as potential inhibitors of microtubule affinity-regulating kinase 4 for the therapeutic management of cell proliferation and metastasis. Eur. J. Med. Chem..

[cit31] Chamani J., Heshmati M. (2008). Mechanism for stabilization of the molten globule state of papain by sodium n-alkyl sulfates: Spectroscopic and calorimetric approaches. J. Colloid Interface Sci..

[cit32] Rehman M. T., Alajmi M. F., Hussain A., Rather G. M., Khan M. A. (2019). High-throughput virtual screening, molecular
dynamics simulation, and enzyme kinetics identified ZINC84525623 as a potential inhibitor of NDM-1. Int. J. Mol. Sci..

[cit33] Mohamed N. A. L., Kuroda Y., Shibukawa A., Nakagawa T., El Gizawy S., Askal H. F., El Kommos M. E. (1999). Binding analysis of Nilvadipine to plasma lipoproteins by capillary electrophoresis-frontal analysis. J. Pharm. Biomed. Anal..

[cit34] Wani T. A., Zargar S., Hussain A. (2022). Spectroscopic, Thermodynamic and Molecular Docking Studies on Molecular Mechanisms of Drug Binding to Proteins. Molecules.

[cit35] Gholami A., Dehghan G., Rashtbari S., Jouyban A. (2022). Exploring the interaction of clonazepam and diazepam with tau protein: Multispectral and molecular docking studies. J. Mol. Struct..

[cit36] Muteeb G., Alshoaibi A., Aatif M., Rehman M. T. M. T., Qayyum M. Z. Z. (2020). Screening marine algae metabolites as high-affinity inhibitors of SARS-CoV-2 main protease (3CLpro): an in silico analysis to identify novel drug candidates to combat COVID-19 pandemic. Appl. Biol. Chem..

[cit37] Shamsi A., Mohammad T., Anwar S., AlAjmi M. F., Hussain A., Rehman M. T., Islam A., Hassan M. I. (2020). Glecaprevir and Maraviroc are high-affinity inhibitors of SARS-CoV-2 main protease: possible implication in COVID-19 therapy. Biosci. Rep..

