# Supplementary material for: Molecular insights into nilvadipine–hemoglobin interactions: conformational dynamics and binding mechanisms
Source: RSC Adv. 2025 Nov 11;15(51):44007–23. doi: 10.1039/d5ra04162g (PMC12604001; doi:10.1039/d5ra04162g)
Supplement: RA-015-D5RA04162G-s001 [file RA-015-D5RA04162G-s001.pdf]

Supplementary Information (SI) for RSC Advances.
This journal is © The Royal Society of Chemistry 2025

Supplementary Information

Molecular Insights into Nilvadipine-Hemoglobin Interactions: Conformational Dynamics and Binding Mechanisms

Mohd Shahnawaz Khan[1], Md. Tabish Rehman[2], Nojood Al-twaijry[1], Nouf Alafaleq[1], Ibrahim Aldobiyan[1], Majed S. Alokail[1], Areej Ali Alzharani[1], Mohammad Arshad[3], Mohammad F AlAjmi[2]

[1]Department of Biochemistry, College of Science, King Saud University, 11451, Riyadh, Saudi Arabia

[2]Department of Pharmacognosy, College of Pharmacy, King Saud University, Riyadh, Saudi Arabia,

[3]College of Applied Medical Science, King Saud University, Riyadh, Saudi Arabia

Corresponding address:

Prof. Mohd Shahnawaz Khan
Department of Biochemistry, College of Science,
King Saud University, Riyadh, KSA
Email: moskhan@ksu.edu.sa

Supplementary Table 1: Post-simulation clustering analysis of Hb–Nilvadipine complex using GROMACS gmx cluster tool with an RMSD cutoff of 0.2 nm.

Cluster	Population (%)	Hydrogen bonding	Hydrophobic interactions	van der Waals interactions	Nilvadipine Orientation
Cluster 1	52%	ASP64<br>ASN68<br>Heme	LYS61<br>Heme	ALA65, ALA79, LEU80, LEU83	Deeply embedded in hydrophobic pocket
Cluster 2	31%	ASN68	ALA82<br>LEU83<br>Heme	LYS61, ASP64, ALA65, ALA79, LEU80	Tilted toward heme ring
Cluster 3	12%	LYS61<br>ASN68	LEU83<br>Heme	ASP64, ALA65, THR67, LEU80	Shallow orientation at pocket entrance
Cluster 4 (<5%)	5%	-	ALA65<br>Heme	LYS61, ASP64, ASN68, ALA79, LEU80, ALA82, LEU83, LEU86	Highly transient poses

Supplementary Table 2: Quenching in the fluorescence intensity of Hb (5 μM) in the presence of varying concentrations of Nilvadipine (0-50 μM) at 298 K.

Wave-length (nm)	0 μM	5 μM	10 μM	15 μM	20 μM	25 μM	30 μM	35 μM	40 μM	45 μM	50 μM
280	650.124	671.144	784.483	816.475	814.7	808.874	799.2	779.903	772.598	740.085	750.953
281	749.289	769.871	897.17	922.324	921.273	916.625	909.022	894.584	888.546	857.431	870.348
282	789.398	808.478	944.512	975.552	976.368	973.068	962.21	947.335	940.641	907.096	923.317
283	762.033	777.557	908.506	938.015	939.387	938.57	927.624	915.179	909.367	877.481	895.149
284	670.957	681.803	795.006	832.133	833.78	832.332	818.988	802.915	798.947	772.405	789.111
285	534.595	540.83	642.538	683.639	685.339	683.458	669.019	650.662	646.262	616.933	635.655
286	385.684	388.767	466.435	509.326	510.94	508.872	493.805	474.412	469.721	442.462	458.751
287	251.62	251.877	301.697	328.731	329.326	327.639	318.514	305.538	303.047	285.031	296.137
288	148.765	147.98	176.435	190.943	190.973	189.337	184.866	176.983	175.481	164.795	171.588
289	82.9234	81.8396	96.2508	103.243	102.886	101.796	99.5491	94.9979	94.0169	88.3583	92.1579
290	49.0597	48.1901	55.167	58.3432	57.7169	57.0442	55.6668	53.088	52.4991	49.2696	51.1522
291	34.7016	34.3065	38.0145	39.5546	38.7415	38.2511	37.2948	35.4425	35.026	32.967	33.8339
292	31.122	30.9121	33.5494	34.4298	33.416	33.2176	32.2161	30.6355	30.1458	28.4721	29.1031
293	31.8455	31.6022	33.8736	34.6977	33.4599	33.2986	32.1994	30.7162	30.1259	28.5342	29.0593
294	33.5544	33.2902	35.3259	36.0184	34.6062	34.5522	33.2851	31.8444	31.1486	29.5435	30.0537
295	35.4658	35.0659	36.8479	37.4477	35.9415	35.8843	34.5215	33.0614	32.2338	30.7364	31.1643
296	37.3547	36.7796	38.3679	38.8219	37.3174	37.2321	35.7452	34.2342	33.3689	31.9074	32.2326
297	39.2242	38.4741	39.8481	40.1862	38.7234	38.5728	36.9228	35.3705	34.4995	32.9964	33.2407
298	41.1146	40.1744	41.2836	41.475	40.0607	39.8539	38.0553	36.4794	35.5858	34.0168	34.2436
299	43.0434	41.9416	42.7593	42.8761	41.4157	41.1197	39.2431	37.607	36.6605	35.0289	35.2515
300	45.0155	43.7727	44.2954	44.3252	42.8385	42.4772	40.4794	38.8077	37.8022	36.0662	36.2731
301	47.1496	45.6611	45.9209	45.913	44.3292	43.8326	41.7591	40.0879	39.0594	37.2104	37.3862
302	49.347	47.6764	47.6668	47.5176	45.7994	45.2613	43.1206	41.4622	40.382	38.4936	38.5877
303	51.6663	49.8418	49.6198	49.3198	47.3816	46.8632	44.6347	42.8821	41.8118	39.9096	39.8619
304	54.0745	52.1492	51.7931	51.2645	49.1989	48.5896	46.2609	44.3784	43.3983	41.4778	41.2383
305	56.6539	54.646	54.1164	53.2531	51.0954	50.4796	48.0161	46.0381	45.0565	43.1371	42.7406
306	59.3194	57.2851	56.445	55.377	53.0918	52.5095	49.8909	47.832	46.7085	44.7902	44.3539
307	62.1335	60.029	58.8327	57.6424	55.2151	54.6231	51.8682	49.6885	48.4494	46.5392	46.0391
308	65.1153	62.8308	61.3689	59.941	57.3989	56.752	53.8684	51.6149	50.2533	48.3623	47.777
309	68.1832	65.606	63.7552	62.2443	59.537	58.8175	55.8696	53.5832	52.0451	50.1233	49.5636
310	71.0751	68.2087	66.1152	64.4519	61.6195	60.7053	57.7874	55.3849	53.7878	51.8077	51.2672
311	73.8169	70.6641	68.4937	66.5644	63.5884	62.5438	59.5494	57.0603	55.4639	53.4752	52.8511
312	76.4164	72.8997	70.7653	68.5584	65.4596	64.2369	61.2329	58.7342	57.0039	55.0781	54.3089
313	78.6962	75.038	72.7083	70.4443	67.215	65.8379	62.8176	60.2696	58.5574	56.4861	55.6232
314	80.9166	77.0521	74.6339	72.3169	68.8435	67.3161	64.3405	61.7003	60.0554	57.9334	56.9621
315	83.2802	79.0995	76.5029	74.2936	70.5276	68.9025	65.7967	63.2831	61.5062	59.3955	58.3404
316	85.7564	81.245	78.422	76.2475	72.24	70.4959	67.4275	65.0199	63.0311	60.8377	59.7719

317	88.2933	83.5529	80.3723	78.1842	74.0326	72.1135	69.1694	66.6114	64.6243	62.2937	61.1582
318	91.0668	85.8326	82.5563	80.1692	75.9918	73.9196	71.0255	68.2695	66.2389	63.8727	62.8091
319	93.8815	88.3989	84.8734	82.3191	78.0737	75.8851	72.9356	70.1051	67.889	65.5509	64.4616
320	96.7476	91.0423	87.3342	84.4652	80.2563	77.9182	75.0372	71.8615	69.751	67.3216	66.1956
321	99.6431	93.689	89.749	86.665	82.5661	79.9813	76.9977	73.7271	71.6275	69.0412	67.9946
322	102.584	96.282	92.2687	89.0408	84.878	82.1373	79.0646	75.7022	73.5713	70.8774	69.8518
323	105.514	99.0734	94.8645	91.4586	87.1807	84.2175	81.1484	77.7265	75.4942	72.7751	71.6169
324	108.382	101.73	97.428	93.7408	89.5434	86.2737	83.2408	79.7391	77.4593	74.5833	73.31
325	111.15	104.307	99.8696	96.0112	91.7254	88.2711	85.2604	81.751	79.2194	76.2348	75.0147
326	113.828	106.737	102.318	98.2887	93.7741	90.2194	87.3359	83.492	80.9907	78.0548	76.5806
327	116.354	109.123	104.619	100.51	95.7909	92.1233	89.2013	85.2629	82.7082	79.7086	78.2622
328	118.645	111.333	106.736	102.658	97.7223	93.9308	90.968	87.0078	84.3323	81.2877	79.8781
329	120.783	113.43	108.62	104.533	99.44	95.7028	92.5994	88.5462	85.7788	82.7447	81.441
330	122.708	115.329	110.327	106.319	101.008	97.2701	94.0014	89.9562	87.1037	84.1657	82.7315
331	124.437	117.099	111.932	107.826	102.52	98.6728	95.2333	91.248	88.2383	85.3979	83.9145
332	126.032	118.696	113.398	109.113	103.882	99.9668	96.3452	92.3819	89.2161	86.446	84.8504
333	127.465	120.057	114.601	110.23	104.988	101.112	97.33	93.3819	90.1634	87.3917	85.6031
334	128.759	121.238	115.747	111.258	105.977	102.01	98.2013	94.2765	91.0468	88.1616	86.191
335	129.933	122.326	116.796	112.129	106.866	102.73	98.9936	95.0989	91.8566	88.7911	86.7076
336	130.913	123.181	117.58	112.916	107.562	103.352	99.6588	95.7604	92.5857	89.3065	87.1885
337	131.689	123.861	118.127	113.483	108.088	103.811	100.195	96.2307	93.1409	89.752	87.548
338	132.211	124.368	118.503	113.807	108.507	104.094	100.496	96.4374	93.3312	89.9952	87.7492
339	132.459	124.541	118.732	114.094	108.913	104.454	100.655	96.5075	93.3723	90.1986	88.0076
340	132.528	124.472	118.682	114.046	108.787	104.322	100.622	96.496	93.2737	90.1769	87.8992
341	132.411	124.365	118.515	113.973	108.852	104.36	100.578	96.3912	93.0107	90.0784	87.8488
342	132.067	124.121	118.237	113.651	108.553	104.037	100.309	96.1005	92.7522	89.7579	87.5747
343	131.533	123.65	117.835	113.217	108.081	103.648	99.9328	95.7647	92.4667	89.3278	87.1925
344	130.919	123.023	117.194	112.604	107.37	103.053	99.3657	95.268	92.0724	88.8183	86.6048
345	130.178	122.284	116.45	111.842	106.53	102.298	98.5861	94.5509	91.4801	88.1391	85.8275
346	129.142	121.372	115.503	110.939	105.544	101.476	97.5743	93.7392	90.8679	87.3704	85.0151
347	127.871	120.239	114.422	109.978	104.526	100.524	96.5296	92.7403	89.8678	86.4777	84.0513
348	126.629	119.099	113.11	108.85	103.296	99.3136	95.3969	91.6217	88.8196	85.4996	82.9455
349	125.204	117.648	111.725	107.584	101.973	98.0266	94.3288	90.4669	87.6856	84.3596	81.8778
350	123.504	116.13	110.271	106.144	100.564	96.6099	93.1681	89.1589	86.5074	83.1487	80.6759
351	121.759	114.412	108.731	104.595	99.0104	95.1335	91.8819	87.8159	85.0994	81.8509	79.3779
352	120.028	112.684	107.041	102.973	97.3576	93.5636	90.4131	86.439	83.9176	80.5181	78.0646
353	118.032	110.741	105.448	101.288	95.8264	92.088	88.9818	84.9428	82.6251	79.0816	76.862
354	115.978	109.015	103.646	99.5887	94.1865	90.461	87.3767	83.3639	81.1677	77.6313	75.4466
355	113.904	107.044	101.774	97.7505	92.4277	88.8658	85.7122	81.8016	79.6414	76.1944	74.0985
356	111.799	105.135	99.9075	95.8955	90.7802	87.1048	84.1702	80.174	78.1839	74.6885	72.8402
357	109.686	103.052	98.0786	93.9509	89.0573	85.3999	82.6351	78.6013	76.5187	73.2599	71.4244

358	107.597	100.902	95.9811	92.0372	87.2816	83.6515	80.899	77.0177	74.8782	71.6985	69.9289
359	105.425	98.7465	94.0199	89.9607	85.6045	81.7961	79.1761	75.489	73.2875	70.1148	68.4629
360	103.194	96.6374	92.0641	87.9842	83.8366	79.8697	77.3886	73.835	71.8055	68.5097	66.8799
361	100.965	94.3998	89.8493	86.0033	81.8767	78.1363	75.5468	72.0835	70.1678	66.9152	65.1366
362	98.6351	92.2417	87.5687	84.0244	79.9152	76.2702	73.6867	70.29	68.4708	65.2087	63.4731
363	96.1791	90.1016	85.4422	81.9211	77.879	74.3052	71.8542	68.5229	66.6765	63.5799	61.7698
364	93.7141	87.8221	83.3026	79.8831	75.8135	72.4801	69.9685	66.6515	64.9174	61.9358	60.0897
365	91.2651	85.5602	81.1127	77.8019	73.7688	70.6279	68.1334	64.8303	63.142	60.2422	58.4363
366	88.7009	83.2814	79.0452	75.6295	71.752	68.5605	66.2184	63.0535	61.37	58.4791	56.7164
367	86.1059	80.9441	76.898	73.4181	69.7614	66.5563	64.3318	61.2752	59.6142	56.7134	55.0088
368	83.6693	78.5671	74.6796	71.2395	67.7713	64.6225	62.5146	59.4954	57.9174	55.022	53.3751
369	81.2569	76.2413	72.4079	69.0671	65.6811	62.7032	60.6734	57.7166	56.1327	53.342	51.7329
370	78.8086	73.9428	70.1317	66.9572	63.6217	60.8216	58.7655	55.9772	54.3828	51.6868	50.0724
371	76.4932	71.6988	67.965	64.9512	61.6431	59.0487	56.9697	54.2874	52.6564	50.1046	48.5166
372	74.2536	69.5226	65.8438	62.9364	59.771	57.29	55.1324	52.5857	51.0476	48.5355	47.1133
373	71.9461	67.3734	63.8208	60.971	57.8622	55.4907	53.2925	50.8659	49.3502	46.909	45.6427
374	69.5969	65.2011	61.8033	59.0323	56.0075	53.6816	51.5275	49.2344	47.7275	45.3659	44.1607
375	67.2673	63.0571	59.8113	57.1329	54.1757	51.8751	49.8713	47.6106	46.0988	43.8311	42.6935
376	64.9808	60.922	57.7572	55.2142	52.3714	50.1173	48.2382	46.0173	44.5276	42.4207	41.2508
377	62.7489	58.7669	55.7281	53.4151	50.4086	48.3894	46.6373	44.4037	42.9481	40.9124	39.7232
378	60.4628	56.6555	53.6605	51.4914	48.5858	46.6114	44.9602	42.7659	41.4034	39.4961	38.1792
379	58.2174	54.5733	51.6825	49.6221	46.751	44.8174	43.2821	41.1632	39.9178	38.0167	36.7174
380	56.0537	52.4054	49.6282	47.6949	44.9407	43.1038	41.5599	39.513	38.3458	36.6223	35.3632
381	53.7774	50.2469	47.6656	45.7179	43.1802	41.3806	39.8172	37.8819	36.8078	35.1569	34.0329
382	51.4945	48.1996	45.7285	43.7785	41.5476	39.5825	38.0981	36.3143	35.2624	33.7932	32.6598
383	49.3059	46.1588	43.8604	41.9627	39.8406	37.8022	36.4715	34.8789	33.7999	32.4748	31.3276
384	47.2254	44.101	41.9122	40.1406	38.1569	36.1544	34.9127	33.4154	32.3359	31.1056	30.033
385	45.1441	42.1879	40.0446	38.386	36.5342	34.495	33.4307	31.9808	30.9064	29.7754	28.748
386	43.1817	40.3511	38.2672	36.6935	34.8682	32.891	32.0121	30.565	29.5201	28.4593	27.4587
387	41.209	38.4583	36.5788	35.023	33.1776	31.419	30.5963	29.2247	28.2114	27.1957	26.2541
388	39.3362	36.6588	34.954	33.4192	31.591	30.0424	29.2611	27.8894	26.9336	25.9171	25.1047
389	37.4894	35.0289	33.3966	31.8824	30.122	28.7027	27.9307	26.5613	25.6886	24.7575	23.9497
390	35.7293	33.4078	31.8902	30.4429	28.7204	27.3859	26.6196	25.3493	24.5525	23.59	22.8259
391	34.0645	31.8116	30.3433	29.0826	27.3983	26.0836	25.3589	24.1675	23.4437	22.4579	21.7588
392	32.4594	30.2938	28.8535	27.7186	26.1674	24.8541	24.1862	23.0221	22.3456	21.3919	20.7508
393	30.9132	28.8027	27.4114	26.4112	24.9638	23.6876	23.0121	21.9137	21.2972	20.3571	19.8024
394	29.4498	27.3334	26.036	25.1058	23.8158	22.5383	21.8601	20.8751	20.2602	19.3657	18.8658
395	27.9677	25.9464	24.7343	23.8514	22.6858	21.4359	20.8149	19.8393	19.2468	18.4423	17.9318
396	26.5332	24.6553	23.513	22.615	21.5241	20.3868	19.7819	18.8174	18.2681	17.5302	17.0644
397	25.1887	23.3778	22.3373	21.4092	20.3454	19.3332	18.7437	17.8416	17.3403	16.643	16.1831
398	23.8327	22.1258	21.1609	20.2514	19.2263	18.2826	17.764	16.9243	16.4319	15.7806	15.3237

399	22.4373	20.9131	20.0223	19.1507	18.1244	17.278	16.8506	16.0095	15.5386	14.9787	14.5176
400	21.2228	19.7704	18.9276	18.1063	17.1344	16.3337	15.9406	15.1848	14.7081	14.1615	13.7184
401	20.0805	18.6707	17.8926	17.159	16.2833	15.4168	15.0489	14.3896	13.9634	13.4342	13.0038
402	18.9889	17.6624	16.9125	16.2757	15.5142	14.5857	14.2504	13.6807	13.2786	12.7499	12.3711
403	18.0537	16.7898	16.0308	15.4949	14.7367	13.8741	13.5476	12.9901	12.5847	12.1237	11.7963
404	17.283	16.0222	15.2567	14.7913	14.0411	13.2182	12.8813	12.3909	12.0568	11.5046	11.2376
405	16.5341	15.3058	14.5961	14.1448	13.417	12.6739	12.3406	11.8628	11.6137	11.0104	10.7813
406	15.8898	14.694	14.0125	13.5581	12.8505	12.2237	11.8725	11.4255	11.1447	10.5518	10.3627
407	15.3632	14.1998	13.5573	13.0761	12.3492	11.8559	11.4944	11.0141	10.7351	10.1896	10.0099
408	14.8814	13.7705	13.1585	12.6573	12.016	11.5021	11.1542	10.7377	10.4733	9.88819	9.69173
409	14.4894	13.3977	12.8384	12.3263	11.7278	11.2289	10.8968	10.5111	10.2004	9.65727	9.4732
410	14.2082	13.1271	12.5835	12.0409	11.4926	10.976	10.6633	10.3141	9.9396	9.46388	9.30412
411	13.9799	12.9169	12.3889	11.8395	11.2978	10.7719	10.4696	10.1541	9.7554	9.30539	9.12743
412	13.8107	12.7303	12.2035	11.7094	11.1639	10.6057	10.3062	10.0007	9.61452	9.15465	8.97404
413	13.7039	12.5805	12.0687	11.5756	11.0146	10.4909	10.1792	9.84965	9.48597	9.02929	8.86701
414	13.6057	12.5039	11.9582	11.49	10.9286	10.3866	10.0636	9.72713	9.36951	8.92476	8.76364
415	13.5231	12.3776	11.8421	11.4298	10.8464	10.3256	9.96191	9.6203	9.27042	8.82557	8.65639
416	13.4723	12.2431	11.7168	11.3518	10.7802	10.2746	9.92796	9.52372	9.18737	8.71601	8.56924
417	13.4005	12.1594	11.6239	11.2635	10.7023	10.2046	9.86997	9.43991	9.12149	8.64146	8.49879
418	13.3397	12.0967	11.5521	11.1804	10.6293	10.1342	9.78437	9.35075	9.05114	8.59768	8.42552
419	13.2867	12.0018	11.4356	11.0661	10.5337	10.074	9.69749	9.26647	8.97526	8.51217	8.36249
420	13.1749	11.9284	11.3297	10.9279	10.4085	9.95978	9.58645	9.17649	8.90233	8.40717	8.29683
421	13.0481	11.8431	11.2274	10.7913	10.2654	9.83939	9.45554	9.05085	8.80341	8.35183	8.19595
422	12.876	11.6837	11.0943	10.7007	10.1595	9.6729	9.32148	8.91847	8.67668	8.24296	8.06448
423	12.6835	11.473	10.9188	10.5689	10.0162	9.52693	9.16061	8.71294	8.53657	8.0895	7.96435
424	12.4615	11.259	10.734	10.3784	9.82458	9.34127	8.99835	8.52558	8.38253	7.93468	7.80947
425	12.2432	11.023	10.5289	10.1497	9.65893	9.12587	8.82239	8.29424	8.21966	7.8008	7.63716
426	11.9896	10.785	10.3138	9.97482	9.48379	8.90811	8.63578	8.11761	8.03589	7.60441	7.51216
427	11.7529	10.5597	10.0871	9.69581	9.22305	8.73289	8.43394	7.90209	7.84927	7.41013	7.36937
428	11.5206	10.3485	9.87156	9.4729	8.99396	8.54103	8.25919	7.77032	7.67487	7.2455	7.15497
429	11.2787	10.0992	9.693	9.28101	8.80665	8.34444	8.0839	7.60619	7.49515	7.11276	7.00583
430	11.0451	9.88076	9.5097	9.14439	8.60743	8.18659	7.91718	7.48245	7.32773	6.96572	6.86707
431	10.8146	9.66948	9.31859	8.92697	8.41713	8.0245	7.75178	7.35042	7.19017	6.83774	6.71588
432	10.6081	9.5025	9.14264	8.73956	8.27426	7.88186	7.61055	7.24369	7.07142	6.7434	6.58453
433	10.4284	9.34587	8.97121	8.56305	8.14455	7.74622	7.4737	7.14106	6.93979	6.64962	6.48347
434	10.2701	9.22275	8.81897	8.40569	8.02386	7.62291	7.34949	7.05223	6.84415	6.55365	6.3603
435	10.1104	9.09333	8.67697	8.27717	7.87195	7.52074	7.24426	6.93085	6.73731	6.44745	6.26441
436	9.9865	8.98956	8.56443	8.16482	7.74048	7.44194	7.13387	6.80592	6.63777	6.34893	6.18312
437	9.87816	8.86598	8.45341	8.06685	7.65402	7.34873	7.02355	6.72771	6.53102	6.25692	6.12709
438	9.73676	8.73817	8.3483	7.96949	7.55805	7.26254	6.94159	6.64696	6.46346	6.17002	6.09146
439	9.60375	8.62332	8.21776	7.8813	7.47617	7.21027	6.87761	6.56043	6.38766	6.13072	6.08136

440	9.52345	8.527	8.12945	7.76915	7.42209	7.1556	6.84524	6.52604	6.35866	6.12444	6.08195
441	9.42488	8.4197	8.03667	7.68746	7.36612	7.09129	6.84271	6.50928	6.34729	6.1022	6.07014
442	9.30531	8.27861	7.97628	7.60772	7.28061	7.03452	6.85248	6.48342	6.33302	6.07131	6.06431
443	9.20586	8.17403	7.92364	7.58906	7.22631	7.00196	6.86284	6.44762	6.33648	6.06897	6.07018
444	9.16145	8.07984	7.86879	7.57473	7.20655	6.96162	6.8609	6.45209	6.3346	6.03327	6.06264
445	9.05312	8.03269	7.81435	7.57132	7.20818	6.93808	6.86139	6.45	6.32512	5.96897	6.05598
446	8.98606	7.97834	7.76886	7.54783	7.17783	6.93459	6.82615	6.42368	6.31265	5.97909	6.04939
447	8.93987	7.95316	7.73894	7.54053	7.18631	6.95724	6.80071	6.4432	6.32298	5.98505	6.03415
448	8.92767	7.93953	7.70476	7.48658	7.17911	6.95692	6.80168	6.4688	6.30458	6.02082	6.01834
449	8.87089	7.92875	7.70346	7.4638	7.14516	6.96996	6.76572	6.47889	6.33822	6.02618	6.02517
450	8.85351	7.889	7.69428	7.45689	7.11723	6.97918	6.74297	6.48598	6.35473	6.05823	6.04282

Supplementary Table 3: Quenching in the fluorescence intensity of Hb (5 μM) in the presence of varying concentrations of Nilvadipine (0-50 μM) at 303 K.

Wave-length (nm)	0 μM	5 μM	10 μM	15 μM	20 μM	25 μM	30 μM	35 μM	40 μM	45 μM	50 μM
280	589.89	748.39	793.163	773.799	773.393	768.355	774.797	771.317	745.587	746.789	727.661
281	676.051	857.93	904.007	888.31	887.75	883.912	889.514	887.039	863.087	864.149	841.465
282	708.621	899.896	951.456	936.038	934.859	931.63	938.235	937.014	912.808	912.357	889.295
283	680.995	864.545	913.307	902.612	899.612	897.873	904.101	904.462	882.84	881.399	859.648
284	597.174	756.75	799.942	792.657	787.525	786.173	791.797	793.376	776.762	775.033	756.44
285	474.541	600.413	647.469	641.075	633.795	631.533	638.259	640.234	621.599	619.563	601.299
286	341.746	430.491	471.315	465.4	456.928	454.142	461.289	463.475	446.147	443.825	431.379
287	222.641	278.301	303.518	302.61	294.784	292.1	297.064	299.003	287.498	285.913	277.88
288	131.995	162.643	176.621	177.504	171.465	168.879	172.091	173.442	166.144	165.127	160.61
289	74.1617	89.3221	96.2874	96.8446	93.1226	91.0008	92.8007	93.3308	89.1302	88.4817	86.1202
290	44.4597	51.9316	55.1762	54.5955	52.929	51.4986	52.303	52.3121	49.8331	49.4299	48.0408
291	32.3733	36.5006	38.0623	36.7084	36.1511	35.073	35.4483	35.1354	33.4024	33.0438	32.1357
292	29.6176	32.5241	33.6226	32.014	31.5823	30.6845	30.8574	30.4265	28.9762	28.6144	27.7411
293	30.4913	33.0768	33.9485	32.2416	31.7413	30.879	31.0257	30.5051	29.0479	28.6863	27.7933
294	32.2741	34.6088	35.2835	33.5641	32.9169	32.1086	32.166	31.6046	30.1139	29.6658	28.7542
295	34.1201	36.2197	36.8139	34.9435	34.215	33.4269	33.3127	32.8152	31.2467	30.7834	29.8529
296	35.9966	37.8037	38.3447	36.3343	35.49	34.671	34.5229	34.0346	32.3666	31.8854	30.9357
297	37.8125	39.3815	39.8167	37.7679	36.8675	35.9474	35.7347	35.1939	33.4542	32.9899	32.0274
298	39.6127	40.9718	41.2671	39.2347	38.2226	37.2367	36.8679	36.2767	34.6054	34.1022	33.0786
299	41.4453	42.525	42.7697	40.6344	39.5815	38.5268	38.0432	37.417	35.7678	35.2825	34.1674
300	43.4294	44.1914	44.2703	42.0762	40.9615	39.8704	39.3673	38.6004	37.0018	36.5009	35.3056
301	45.5081	45.9999	45.8443	43.6108	42.3908	41.2633	40.7288	39.8542	38.2557	37.7824	36.4978
302	47.699	47.8524	47.5009	45.1646	43.8926	42.7284	42.1405	41.2266	39.5994	39.0457	37.7881
303	50.0056	49.823	49.2162	46.7947	45.5365	44.329	43.7463	42.7484	41.0337	40.3975	39.2264
304	52.4754	52.0608	51.076	48.6484	47.3493	46.0513	45.4669	44.3342	42.551	41.8803	40.7049
305	55.102	54.4023	53.1404	50.6956	49.2582	47.9544	47.2189	46.0373	44.1655	43.4175	42.2265
306	57.852	56.7928	55.3965	52.8053	51.3166	49.9912	49.0719	47.8143	45.9211	45.0498	43.8825
307	60.7765	59.4026	57.7997	55.085	53.4748	52.0973	51.0722	49.673	47.7262	46.8731	45.6506
308	63.7685	62.1184	60.3116	57.497	55.6655	54.249	53.0589	51.594	49.5707	48.7208	47.3651
309	66.7321	64.7389	62.7807	59.8356	57.8112	56.4153	55.0176	53.4495	51.4809	50.4286	49.1017
310	69.5391	67.2378	65.1177	61.9418	59.8787	58.3699	56.9712	55.2025	53.2995	52.1445	50.7936
311	72.1497	69.6586	67.3073	64.0156	61.8595	60.2474	58.8192	56.8723	54.95	53.8097	52.3859
312	74.6392	71.9175	69.303	65.9892	63.6593	61.9637	60.4926	58.4557	56.514	55.3054	53.854
313	76.9746	73.9161	71.1778	67.7589	65.2616	63.6404	62.1053	59.9278	58.0167	56.7119	55.2129
314	79.1862	75.8969	73.0106	69.5548	66.9168	65.2355	63.6946	61.4516	59.4155	58.1624	56.4879

315	81.485	77.9889	75.0113	71.4149	68.7066	66.9693	65.2845	62.9779	60.8823	59.5894	57.8775
316	83.9277	80.2456	77.0145	73.3056	70.4909	68.6456	66.9235	64.5691	62.3765	61.0774	59.2427
317	86.364	82.3819	79.0726	75.1808	72.3377	70.4214	68.6627	66.2377	63.9095	62.6222	60.7183
318	88.9329	84.8386	81.1287	77.2205	74.3513	72.1538	70.3764	67.9953	65.5556	64.2633	62.395
319	91.6434	87.2781	83.4649	79.2966	76.4451	74.1674	72.2804	69.8182	67.3452	66.0119	64.1992
320	94.3636	89.6548	85.7198	81.5795	78.5494	76.0358	74.3026	71.7791	69.1875	67.7547	66.0324
321	97.0748	92.0317	88.0518	83.8212	80.5956	78.1291	76.2895	73.8087	71.1357	69.6207	67.8952
322	99.8615	94.7008	90.4259	86.2327	82.7233	80.446	78.2932	75.8254	73.0401	71.567	69.7075
323	102.729	97.2107	92.9335	88.6316	84.9383	82.5852	80.4134	77.7437	74.95	73.5637	71.5343
324	105.49	99.8499	95.2968	90.9771	87.0625	84.6745	82.4943	79.7333	76.7943	75.4267	73.2563
325	108.204	102.476	97.6112	93.2025	89.1318	86.9077	84.396	81.5868	78.585	77.3573	74.9401
326	110.873	104.984	99.9085	95.467	91.3106	88.9926	86.3096	83.2834	80.3249	79.0761	76.6402
327	113.516	107.297	102.239	97.5876	93.4309	90.811	88.0511	84.9628	82.093	80.6576	78.1838
328	115.909	109.529	104.489	99.5049	95.4236	92.7483	89.7524	86.6908	83.6855	82.1321	79.6332
329	118.173	111.513	106.482	101.279	97.2083	94.4168	91.291	88.2114	85.1342	83.5967	81.0577
330	120.315	113.401	108.32	103.007	98.8141	95.9859	92.7469	89.6387	86.5207	84.9098	82.2852
331	122.275	115.204	109.996	104.483	100.229	97.4314	94.1058	91.012	87.7691	86.1708	83.4477
332	123.881	116.794	111.472	105.802	101.536	98.7121	95.4024	92.1623	88.8444	87.2927	84.559
333	125.289	118.094	112.687	107.036	102.562	99.7702	96.4624	93.1344	89.7062	88.2404	85.4879
334	126.52	119.22	113.851	108.139	103.488	100.683	97.3298	93.9289	90.4508	88.9781	86.253
335	127.54	120.157	114.81	108.996	104.319	101.4	98.0794	94.6157	91.0153	89.5527	86.9029
336	128.321	120.904	115.511	109.705	104.984	101.929	98.736	95.0814	91.5429	89.9367	87.3482
337	128.968	121.535	115.981	110.249	105.425	102.301	99.2687	95.4218	91.958	90.3011	87.665
338	129.432	122.000	116.314	110.689	105.753	102.557	99.6818	95.6721	92.3632	90.6076	87.9031
339	129.753	122.442	116.442	110.94	105.986	102.716	100.041	95.7886	92.8382	90.5163	87.8486
340	129.905	122.672	116.527	111.022	105.966	102.709	99.9352	95.6889	92.6901	90.6674	87.8172
341	129.845	122.625	116.469	111.007	105.84	102.589	99.9213	95.5999	92.7092	90.5554	87.6585
342	129.534	122.405	116.217	110.788	105.538	102.342	99.6152	95.3592	92.3655	90.2597	87.3944
343	129.048	122.006	115.802	110.348	105.21	101.991	99.121	94.9094	91.868	89.8544	86.8623
344	128.39	121.317	115.283	109.749	104.662	101.428	98.5243	94.3221	91.1589	89.3186	86.312
345	127.504	120.491	114.388	108.933	103.997	100.771	97.7571	93.6589	90.4355	88.5817	85.7017
346	126.536	119.495	113.447	108.052	103.116	99.8528	96.8072	92.786	89.6057	87.8138	84.8844
347	125.436	118.302	112.29	106.938	102.164	98.7995	95.7678	91.7229	88.7489	86.832	83.936
348	124.205	116.996	111.123	105.801	100.924	97.6623	94.712	90.6797	87.7843	85.8058	82.9606
349	122.744	115.734	109.697	104.578	99.6243	96.3763	93.3879	89.5435	86.8251	84.6726	81.8025
350	121.187	114.249	108.278	103.206	98.1944	94.848	92.0831	88.3649	85.5586	83.415	80.5967
351	119.522	112.617	106.659	101.69	96.7568	93.4208	90.5789	87.0609	84.2415	82.034	79.3023
352	117.899	111.079	105.171	100.121	95.1944	92.0676	89.2161	85.7693	82.8449	80.6595	78.0093
353	116.18	109.443	103.529	98.4901	93.7835	90.5236	87.6977	84.2629	81.4805	79.1849	76.7868

354	114.445	107.568	101.806	96.8023	92.2161	88.9911	86.2432	82.7649	79.9612	77.8258	75.502
355	112.543	105.772	100.044	95.0578	90.5453	87.5466	84.6932	81.1636	78.5145	76.4471	74.0592
356	110.586	103.983	98.3117	93.1995	88.9143	85.9347	83.3221	79.618	77.0514	75.1008	72.6171
357	108.511	101.944	96.4216	91.4168	87.2956	84.1798	81.6689	78.0379	75.5991	73.711	71.1725
358	106.336	99.8905	94.3952	89.5525	85.4374	82.4877	80.0226	76.4456	73.9817	72.3055	69.6593
359	104.068	97.7462	92.4343	87.6339	83.705	80.762	78.3177	74.8027	72.362	70.7208	68.1006
360	101.843	95.537	90.5014	85.7422	81.9211	78.9599	76.5452	73.1511	70.6814	69.1378	66.5455
361	99.5478	93.3579	88.4986	83.8839	80.0814	77.1543	74.7048	71.4051	68.8901	67.4545	64.9451
362	97.1673	91.1297	86.462	81.9025	78.1206	75.2824	72.8117	69.634	67.1155	65.7768	63.2081
363	94.7807	88.9143	84.4379	79.7742	76.1908	73.4239	70.8846	67.8456	65.412	64.0567	61.4833
364	92.4034	86.7427	82.3637	77.7178	74.2061	71.5769	69.0094	66.056	63.683	62.358	59.7835
365	89.9596	84.5073	80.2541	75.6944	72.284	69.7066	67.1794	64.26	61.9216	60.6128	58.0784
366	87.4768	82.1657	78.0433	73.5443	70.2927	67.8109	65.3481	62.4676	60.2098	58.841	56.4094
367	84.9809	79.8433	75.8222	71.4085	68.2943	65.8752	63.5421	60.699	58.4718	57.0412	54.8266
368	82.5554	77.5576	73.6632	69.3873	66.2768	63.9727	61.7658	58.9777	56.7243	55.2792	53.2
369	80.1499	75.2536	71.5439	67.339	64.2446	62.0871	59.9351	57.2854	55.0025	53.5671	51.5286
370	77.7631	72.9714	69.3979	65.2825	62.2592	60.1444	58.0575	55.5564	53.324	51.8616	49.9272
371	75.518	70.7694	67.3017	63.3417	60.3162	58.2974	56.2315	53.838	51.6905	50.2516	48.3908
372	73.3389	68.6339	65.2584	61.3558	58.5022	56.525	54.5143	52.145	50.1034	48.7269	46.8126
373	71.1346	66.4399	63.222	59.4674	56.7928	54.6785	52.8449	50.4893	48.4906	47.2072	45.2783
374	68.8792	64.3069	61.1632	57.544	55.0554	52.83	51.2021	48.7459	46.8557	45.6783	43.8887
375	66.6887	62.2605	59.0817	55.7019	53.2992	51.1484	49.5027	47.175	45.3009	44.2432	42.4148
376	64.3393	60.2409	57.0725	53.8244	51.5842	49.4262	47.8732	45.6288	43.7086	42.8234	41.0053
377	62.0071	58.2492	55.0943	52.0563	49.7569	47.7118	46.2172	44.0889	42.1428	41.3637	39.5684
378	59.6693	56.2363	53.1212	50.132	47.8591	45.9877	44.4855	42.4893	40.5899	39.8366	38.1418
379	57.3942	54.1727	51.1604	48.2582	45.9987	44.3345	42.8335	40.956	39.1591	38.3571	36.6509
380	55.128	52.0899	49.2609	46.3256	44.1413	42.5654	41.2507	39.2883	37.7112	36.8178	35.2388
381	52.9954	50.0185	47.2701	44.462	42.3502	40.8338	39.6123	37.7034	36.3324	35.2414	33.7631
382	50.8308	47.8078	45.2856	42.5943	40.6438	39.1665	37.9223	36.1293	34.845	33.7523	32.3787
383	48.7423	45.7979	43.3778	40.8415	38.9704	37.5671	36.3436	34.6065	33.4331	32.3625	31.0578
384	46.7155	43.8067	41.5513	39.0812	37.3685	35.9005	34.7643	33.1339	32.0191	30.9516	29.7613
385	44.752	41.8316	39.7461	37.355	35.8076	34.3904	33.2439	31.735	30.5947	29.6295	28.4693
386	42.7505	39.9106	38.0694	35.7125	34.2277	32.9226	31.7454	30.3181	29.2121	28.3629	27.2317
387	40.8495	38.2086	36.3954	34.1388	32.7208	31.4631	30.3952	28.9725	27.9583	27.1237	26.0138
388	38.9643	36.5052	34.7148	32.5465	31.2561	30.0667	29.0304	27.7296	26.6672	25.9114	24.8102
389	37.1695	34.8533	33.0923	31.0219	29.7907	28.749	27.7164	26.4638	25.4018	24.7429	23.6539
390	35.44	33.2791	31.5645	29.6167	28.4035	27.436	26.4263	25.2181	24.2957	23.6046	22.566
391	33.7832	31.7395	30.0891	28.2387	27.1163	26.193	25.1841	24.0131	23.2203	22.5156	21.5364
392	32.1751	30.2827	28.6871	26.8997	25.8683	24.9874	23.9932	22.8659	22.1238	21.4387	20.5619

393	30.6547	28.867	27.343	25.6396	24.6523	23.8181	22.8872	21.7565	21.0821	20.4219	19.554
394	29.1563	27.5222	26.0468	24.4475	23.5177	22.6449	21.7832	20.7475	20.0898	19.4953	18.6104
395	27.7322	26.2203	24.7978	23.2927	22.4082	21.5136	20.771	19.7928	19.0791	18.599	17.7278
396	26.3416	24.9527	23.5482	22.1286	21.2514	20.3909	19.749	18.8525	18.0945	17.7047	16.8684
397	24.9986	23.6255	22.3228	21.0153	20.1338	19.3146	18.7395	17.8786	17.1357	16.816	16.0157
398	23.6234	22.3615	21.1458	19.936	19.0704	18.2919	17.7701	16.922	16.2552	15.9383	15.2067
399	22.3306	21.2001	19.9862	18.8815	18.055	17.3646	16.8601	16.0267	15.3958	15.0466	14.4477
400	21.048	20.0953	18.8984	17.8159	17.0742	16.4603	15.9663	15.1345	14.5756	14.2064	13.7057
401	19.8638	19.0939	17.8631	16.8493	16.1593	15.6065	15.1474	14.3681	13.8212	13.4801	12.9622
402	18.8069	18.2275	16.9518	15.9168	15.281	14.8004	14.3965	13.6598	13.1812	12.8197	12.3088
403	17.8918	17.4242	16.1298	15.1166	14.5169	14.0969	13.6648	12.9836	12.5342	12.1817	11.7325
404	17.0757	16.7315	15.4379	14.3692	13.8182	13.4539	13.0507	12.4021	12.0038	11.6358	11.154
405	16.327	16.1109	14.7376	13.7776	13.2441	12.8683	12.471	11.8955	11.4996	11.1761	10.6466
406	15.7125	15.5651	14.1984	13.2279	12.7572	12.351	11.965	11.3899	11.0739	10.7553	10.2466
407	15.1434	15.1191	13.7089	12.7985	12.3631	11.9567	11.5343	11.0057	10.6761	10.3844	9.90702
408	14.7238	14.7439	13.2764	12.4015	12.0003	11.572	11.1966	10.7034	10.3814	10.1349	9.600
409	14.3797	14.4332	12.9322	12.1181	11.6954	11.2694	10.9025	10.4071	10.1129	9.90369	9.41151
410	14.1455	14.207	12.7006	11.8641	11.4466	11.042	10.6883	10.2023	9.9054	9.68816	9.23626
411	13.9463	14.0206	12.4814	11.6663	11.2627	10.8605	10.5205	10.0223	9.72115	9.48421	9.08134
412	13.7947	13.8625	12.3155	11.4902	11.0882	10.6844	10.3437	9.86919	9.56186	9.35739	8.92347
413	13.6574	13.7496	12.1809	11.3746	10.9731	10.5702	10.223	9.73242	9.4484	9.23841	8.83433
414	13.5305	13.6159	12.0376	11.2706	10.8758	10.4663	10.0992	9.62617	9.35675	9.14109	8.70729
415	13.4049	13.4755	11.929	11.1681	10.7749	10.3904	9.99755	9.54825	9.27386	9.04195	8.60839
416	13.3029	13.3484	11.8186	11.0813	10.6719	10.3492	9.91104	9.51009	9.20272	8.95156	8.53323
417	13.2256	13.2084	11.7199	11.002	10.5915	10.2964	9.85513	9.48453	9.16452	8.84562	8.46687
418	13.1399	13.0517	11.6236	10.9202	10.4995	10.2188	9.78613	9.43593	9.09203	8.72587	8.36039
419	13.0468	12.8915	11.5369	10.8357	10.4167	10.1028	9.72262	9.36536	8.97806	8.62173	8.2923
420	12.9568	12.7111	11.4593	10.7478	10.3233	9.99144	9.65153	9.2688	8.87027	8.54848	8.20515
421	12.8585	12.5209	11.334	10.6331	10.1858	9.84457	9.57166	9.17247	8.77661	8.46693	8.08531
422	12.7211	12.3243	11.1787	10.4954	10.0461	9.69819	9.44544	9.03308	8.61206	8.36786	7.97413
423	12.5519	12.0616	10.9949	10.3216	9.87647	9.54093	9.27833	8.86838	8.4386	8.25119	7.84755
424	12.3603	11.7887	10.8348	10.1318	9.64714	9.38572	9.08398	8.69692	8.2872	8.08011	7.68167
425	12.1442	11.5703	10.5987	9.95558	9.44309	9.1968	8.89764	8.53178	8.11515	7.911	7.50866
426	11.8847	11.2991	10.4113	9.77477	9.27282	9.00454	8.69534	8.34497	7.92914	7.73248	7.33224
427	11.6363	11.0119	10.2023	9.58916	9.05197	8.79676	8.50086	8.18703	7.74655	7.55194	7.16197
428	11.3979	10.7844	9.99992	9.36832	8.84741	8.60824	8.30521	8.0337	7.58201	7.38209	6.9929
429	11.1767	10.5785	9.78035	9.14514	8.68744	8.43133	8.14717	7.89025	7.4019	7.24353	6.8615
430	10.9597	10.3344	9.57674	8.92709	8.49646	8.2577	7.94314	7.74404	7.26833	7.10784	6.71953
431	10.7607	10.1576	9.36364	8.74051	8.30438	8.10263	7.77415	7.60442	7.11562	6.98573	6.61199

432	10.5691	10.0129	9.18933	8.56623	8.16786	7.96332	7.62835	7.45865	6.99772	6.87148	6.47443
433	10.3882	9.87215	9.03322	8.43248	8.04826	7.82326	7.50995	7.35071	6.88182	6.76652	6.37288
434	10.2132	9.72688	8.86416	8.31392	7.9358	7.67998	7.35923	7.23575	6.77321	6.66165	6.23571
435	10.0642	9.60549	8.72773	8.18743	7.82936	7.55294	7.25066	7.13885	6.66495	6.54272	6.14837
436	9.91385	9.48591	8.60765	8.0455	7.74193	7.44623	7.15087	7.06696	6.58244	6.43769	6.07794
437	9.76653	9.38314	8.5157	7.91667	7.65375	7.34579	7.05982	7.00145	6.5096	6.3536	6.02411
438	9.60964	9.29893	8.42038	7.80258	7.56837	7.26329	7.00071	6.93404	6.45391	6.29191	5.97569
439	9.47913	9.224	8.30407	7.722	7.50175	7.22892	6.95499	6.87943	6.39251	6.25714	5.95431
440	9.35278	9.14872	8.23928	7.63984	7.44596	7.19227	6.90494	6.83356	6.34514	6.23633	5.92249
441	9.23698	9.07849	8.16855	7.58322	7.40178	7.15214	6.85392	6.78938	6.3286	6.21953	5.88586
442	9.14945	9.0028	8.08407	7.53805	7.3757	7.12352	6.81774	6.74811	6.30045	6.22253	5.88793
443	9.0915	8.93421	8.04615	7.4845	7.35514	7.09526	6.76345	6.73681	6.30292	6.21461	5.86297
444	9.00741	8.88809	8.03071	7.40913	7.29376	7.06737	6.72759	6.77254	6.30312	6.21055	5.85142
445	8.95169	8.8217	7.96566	7.37629	7.2662	7.04399	6.70208	6.77949	6.2847	6.192	5.83915
446	8.89285	8.78821	7.93638	7.34728	7.23643	7.04363	6.70081	6.78828	6.25301	6.20104	5.86767
447	8.83092	8.76262	7.92696	7.35932	7.18063	7.04138	6.68595	6.81464	6.27888	6.19873	5.87811
448	8.77626	8.72506	7.9051	7.41179	7.16925	7.05488	6.68546	6.85171	6.29524	6.22455	5.91726
449	8.73866	8.65851	7.90703	7.43231	7.1821	7.07311	6.70723	6.83886	6.32525	6.22823	5.95655
450	8.69108	8.63256	7.92048	7.43665	7.15379	7.0748	6.73556	6.84777	6.38172	6.27919	6.00104

Supplementary Table 4: Quenching in the fluorescence intensity of Hb (5 μM) in the presence of varying concentrations of Nilvadipine (0-50 μM) at 310 K.

Wave-length (nm)	0 μM	5 μM	10 μM	15 μM	20 μM	25 μM	30 μM	35 μM	40 μM	45 μM	50 μM
280	628.488	765.491	815.246	807.593	806.072	790.291	782.371	770.144	744.482	728.891	718.775
281	726.692	883.101	922.74	917.11	915.954	903.941	897.662	888.563	866.732	853.838	844.37
282	769.358	943.54	984.241	980.907	980.314	971.58	965.09	954.592	929.796	917.222	906.034
283	746.509	921.345	957.859	957.613	957.089	950.286	944.132	934.939	910.964	900.656	889.299
284	661.108	818.637	862.693	862.581	861.716	851.731	843.285	831.63	811.743	805.286	794.841
285	530.929	671.317	721.601	721.414	720.469	708.665	698.971	685.774	663.213	656.502	644.483
286	387.011	498.429	551.487	551.321	550.3	537.374	527.151	513.2	489.167	482.368	470.344
287	254.893	327.776	365.712	365.428	364.315	352.8	345.292	336.453	320.466	316.859	308.453
288	152.345	194.301	215.862	215.691	214.95	208.059	203.441	198.12	188.349	187.004	181.597
289	85.8139	106.794	117.774	117.32	116.768	112.825	110.357	107.257	101.893	101.314	98.138
290	50.5536	60.6122	65.655	65.1443	64.4983	62.1485	60.781	58.9991	56.0963	55.6413	53.8559
291	35.2621	40.3303	42.8223	42.1136	41.3697	39.9627	38.9674	37.669	36.0245	35.5088	34.3243
292	31.0584	34.3217	35.9482	35.1763	34.3981	33.2876	32.4604	31.229	29.9571	29.3208	28.4058
293	31.4319	34.3144	35.5921	34.7187	33.9991	32.8219	32.0095	30.7142	29.5623	28.84	27.9515
294	33.0178	35.7337	36.7843	35.7654	35.0973	33.8732	32.9864	31.6408	30.4732	29.6391	28.8082
295	34.7509	37.3047	38.1993	37.0121	36.3952	35.0933	34.0982	32.7245	31.5293	30.646	29.8201
296	36.5201	38.8321	39.6055	38.2984	37.7025	36.2946	35.2309	33.821	32.5883	31.6516	30.8096
297	38.227	40.3265	40.9974	39.562	38.9157	37.4616	36.2721	34.8486	33.664	32.6711	31.8199
298	39.9398	41.786	42.4085	40.7905	40.0741	38.5995	37.3142	35.8733	34.7048	33.661	32.8116
299	41.73	43.2714	43.7899	42.0317	41.3134	39.7522	38.4527	36.9553	35.825	34.6972	33.8222
300	43.6001	44.8103	45.2314	43.3072	42.6003	40.9807	39.659	38.1087	37.0022	35.7518	34.852
301	45.5672	46.4067	46.78	44.6892	43.923	42.3232	40.8104	39.331	38.1929	36.8818	35.9675
302	47.6854	48.1453	48.3754	46.1391	45.307	43.7198	42.107	40.6621	39.4051	38.1117	37.137
303	49.914	50.0337	50.0599	47.6873	46.8155	45.1862	43.5979	42.1217	40.7837	39.4515	38.4476
304	52.2337	52.1161	51.966	49.4539	48.4732	46.8129	45.1924	43.6299	42.3124	40.921	39.8582
305	54.708	54.375	54.0614	51.3903	50.3041	48.544	46.895	45.2314	43.8975	42.4603	41.3624
306	57.3284	56.7717	56.2447	53.3967	52.3024	50.3875	48.7886	46.9248	45.5937	44.0887	42.9632
307	60.0595	59.3286	58.5892	55.5948	54.3917	52.3962	50.7796	48.7583	47.4579	45.8069	44.6698
308	62.9506	61.9291	61.0381	57.915	56.5347	54.4567	52.7341	50.7415	49.2996	47.6593	46.3984
309	65.7915	64.5006	63.4119	60.1563	58.6469	56.5756	54.6886	52.7148	51.0642	49.451	48.102
310	68.4838	66.8728	65.6925	62.3	60.6103	58.5514	56.6068	54.6227	52.7756	51.2206	49.783
311	71.0156	69.2663	67.8774	64.3805	62.4595	60.3868	58.36	56.4878	54.4483	52.936	51.3554
312	73.4403	71.515	69.9888	66.3162	64.278	62.1963	60.1053	58.1311	55.9762	54.564	52.8889
313	75.5639	73.6339	71.929	68.1379	66.0055	63.865	61.6827	59.6201	57.3677	55.9903	54.2652
314	77.585	75.5694	73.7754	69.9354	67.7029	65.3396	63.1725	61.1841	58.7543	57.3587	55.6172

315	79.7032	77.5388	75.5649	71.6951	69.3725	66.9278	64.7088	62.7595	60.2146	58.7042	56.9146
316	81.8384	79.6608	77.5251	73.5337	71.1469	68.5928	66.3363	64.2557	61.6577	60.1159	58.3558
317	84.0004	81.7584	79.5932	75.3104	72.9704	70.1442	67.8632	65.8629	63.0986	61.4787	59.7199
318	86.4821	83.9352	81.6266	77.071	74.7675	71.9415	69.6122	67.5738	64.6794	62.9455	61.1796
319	89.1133	86.2039	83.8887	79.0503	76.6862	73.8777	71.4907	69.3042	66.4448	64.6851	62.7896
320	91.6943	88.7455	86.2558	81.3259	78.7885	76.031	73.4406	71.0877	68.2111	66.4543	64.4509
321	94.3516	91.1728	88.706	83.4331	80.87	78.1722	75.4914	72.9824	70.0899	68.3039	66.13
322	97.2151	93.7109	90.9356	85.7376	82.9753	80.2748	77.5589	74.9443	72.0438	70.1592	67.93
323	99.9743	96.2182	93.3839	88.0992	85.2081	82.3245	79.628	76.8064	74.0099	71.9983	69.8532
324	102.653	98.8559	95.7846	90.3302	87.3316	84.4386	81.6404	78.5777	75.8531	73.7791	71.7235
325	105.247	101.285	98.1293	92.3415	89.4242	86.2566	83.6513	80.4493	77.6227	75.5535	73.4965
326	107.885	103.704	100.253	94.5111	91.5239	88.0441	85.5888	82.2211	79.3084	77.1734	75.0914
327	110.174	105.983	102.453	96.5046	93.4342	89.9526	87.4722	83.8953	81.0339	78.8377	76.6224
328	112.32	108.225	104.538	98.4295	95.2598	91.7527	89.1949	85.6001	82.6251	80.4471	78.0299
329	114.356	110.234	106.448	100.323	97.0165	93.3708	90.8589	87.2684	84.1154	81.8221	79.2636
330	116.329	112.127	108.166	102.088	98.5149	95.0312	92.2629	88.6775	85.5105	83.0919	80.5031
331	118.019	113.781	109.742	103.676	99.9818	96.5174	93.5024	89.9996	86.8732	84.2702	81.676
332	119.6	115.262	111.136	105.043	101.361	97.7001	94.6368	91.122	88.0507	85.3269	82.6541
333	120.958	116.494	112.249	106.218	102.48	98.7609	95.6423	92.077	89.0559	86.2264	83.4368
334	122.082	117.576	113.111	107.128	103.39	99.6573	96.4619	92.8047	89.8749	86.9506	84.1639
335	123.031	118.527	113.896	107.867	104.198	100.328	97.1939	93.3777	90.623	87.56	84.7932
336	123.862	119.23	114.537	108.352	104.792	100.829	97.7972	93.8515	91.0521	87.995	85.2043
337	124.519	119.758	114.969	108.733	105.303	101.308	98.2848	94.2463	91.2965	88.2558	85.5324
338	124.977	120.135	115.289	109.053	105.665	101.609	98.6577	94.4458	91.4746	88.4397	85.7469
339	125.293	120.390	115.670	109.232	105.811	101.767	98.7985	94.5741	91.5640	88.4908	85.7601
340	125.443	120.356	115.589	109.217	105.742	101.738	98.7272	94.5562	91.5632	88.4012	85.638
341	125.334	120.239	115.6	109.04	105.664	101.522	98.5964	94.4909	91.5507	88.2091	85.6099
342	124.94	119.915	115.353	108.811	105.22	101.227	98.2707	94.2594	91.3655	87.9387	85.3675
343	124.488	119.579	114.871	108.301	104.801	100.775	97.7912	93.8872	90.9877	87.5076	84.8998
344	123.841	118.952	114.225	107.633	104.397	100.203	97.1687	93.3566	90.371	87.0582	84.3485
345	122.961	118.029	113.372	106.828	103.669	99.4965	96.5111	92.6805	89.5438	86.452	83.7514
346	121.912	117.05	112.286	105.941	102.769	98.7069	95.5851	91.8994	88.5891	85.7388	82.8826
347	120.634	115.917	111.149	104.896	101.752	97.7502	94.5435	90.8572	87.5372	84.7778	82.0217
348	119.208	114.488	109.947	103.688	100.544	96.5273	93.4491	89.8235	86.4546	83.7415	81.1158
349	117.641	113.032	108.526	102.398	99.3073	95.2828	92.2996	88.7051	85.3781	82.5833	80.1124
350	116.036	111.582	107.075	100.989	97.9308	93.958	90.96	87.4296	84.1574	81.2433	78.975
351	114.347	109.904	105.568	99.5174	96.4358	92.5454	89.563	86.0167	82.8076	79.9646	77.7751
352	112.598	108.184	103.938	97.8632	94.8715	90.953	88.1914	84.7284	81.4773	78.7223	76.4738
353	110.827	106.357	102.325	96.2195	93.2181	89.4985	86.6031	83.3581	80.0545	77.3659	75.0701

354	108.891	104.631	100.633	94.524	91.4415	87.9532	85.0333	81.8367	78.6042	75.943	73.6218
355	106.931	102.795	98.8196	92.7662	89.7476	86.2896	83.5378	80.3233	77.0634	74.6326	72.2065
356	104.862	101.052	97.0259	91.0845	88.057	84.6378	82.0771	78.7285	75.608	73.1767	70.7716
357	103.077	99.2075	95.2421	89.3804	86.3717	83.0376	80.374	77.0813	74.1296	71.7322	69.3401
358	101.029	97.3948	93.2017	87.6948	84.6522	81.2717	78.6676	75.3387	72.647	70.2566	67.9417
359	99.0685	95.4213	91.2313	85.8012	82.8156	79.4387	76.8744	73.7025	71.0114	68.6791	66.4555
360	96.9974	93.4526	89.2991	84.0093	80.8988	77.6221	75.1071	71.9917	69.4573	67.1131	64.906
361	94.8721	91.3121	87.3124	81.979	78.9314	75.8137	73.1484	70.3339	67.8008	65.4865	63.383
362	92.5224	89.1115	85.1634	79.89	76.9831	73.9487	71.3052	68.6592	66.0774	63.7857	61.7508
363	90.2851	86.8938	83.0559	77.7946	75.1164	72.1582	69.5721	66.9	64.281	62.1243	60.0946
364	87.9898	84.6885	80.8989	75.8531	73.2178	70.3203	67.8107	65.1217	62.5886	60.5274	58.5147
365	85.6553	82.4578	78.6884	73.7842	71.2277	68.4422	65.9257	63.3699	60.8692	58.8438	56.8804
366	83.3388	80.2319	76.4289	71.6847	69.2024	66.5438	64.1333	61.5565	59.163	57.1491	55.2401
367	81.0142	78.0028	74.269	69.6368	67.2331	64.6545	62.3683	59.7623	57.4471	55.5183	53.6216
368	78.7097	75.7797	72.1781	67.5449	65.1905	62.7506	60.5388	58.0524	55.8019	53.9104	52.0634
369	76.4498	73.5114	70.1024	65.4274	63.2533	60.8189	58.761	56.3393	54.0979	52.303	50.4671
370	74.1835	71.1568	68.0853	63.3368	61.3755	58.9937	56.9661	54.623	52.4514	50.7415	48.9203
371	71.954	68.9462	66.0891	61.3991	59.5811	57.1454	55.2212	52.9114	50.8363	49.2634	47.4296
372	69.9017	66.793	64.1119	59.5838	57.676	55.2877	53.5336	51.2262	49.3148	47.6884	46.0103
373	67.8339	64.6702	62.0803	57.8406	55.8402	53.4963	51.7683	49.5968	47.7524	46.1252	44.5882
374	65.7135	62.4758	60.0776	56.0739	53.9735	51.7956	50.1265	47.9538	46.2022	44.5639	43.2207
375	63.5652	60.5945	58.083	54.3042	52.228	50.0867	48.4976	46.4361	44.621	43.0088	41.8225
376	61.5528	58.5853	56.1209	52.5583	50.4557	48.3705	46.8894	44.9307	43.0619	41.4582	40.3139
377	59.3882	56.5147	54.1218	50.7001	48.7311	46.6815	45.2024	43.2678	41.4686	40.0173	38.8445
378	57.2483	54.4418	52.1791	48.7486	46.9618	44.996	43.6156	41.6574	39.9562	38.6338	37.4587
379	55.0984	52.5477	50.2157	46.9208	45.2398	43.2731	41.9538	40.148	38.5207	37.2257	36.0437
380	53.0388	50.3992	48.2482	45.1123	43.5205	41.6031	40.332	38.5259	37.0765	35.931	34.6495
381	50.9212	48.3011	46.3007	43.2765	41.8021	39.9648	38.6884	36.9877	35.6169	34.5431	33.3303
382	48.8545	46.3494	44.3902	41.4717	40.1014	38.3008	37.1214	35.5914	34.1618	33.1879	32.0091
383	46.742	44.412	42.5305	39.7887	38.4411	36.6595	35.5928	34.1285	32.7355	31.8265	30.6477
384	44.8466	42.4742	40.7551	38.0475	36.8257	35.0842	34.0828	32.6617	31.3182	30.5414	29.3351
385	42.919	40.5984	39.0329	36.3384	35.1773	33.5578	32.5592	31.3091	29.9989	29.1544	28.0333
386	41.0173	38.8616	37.3074	34.6928	33.6287	32.0789	31.1579	29.9216	28.7018	27.8805	26.7774
387	39.2124	37.1553	35.6746	33.2077	32.1922	30.6811	29.8151	28.5846	27.4454	26.631	25.5748
388	37.5084	35.5289	34.1523	31.7614	30.751	29.3244	28.4918	27.308	26.1814	25.4223	24.4669
389	35.7924	33.8907	32.5881	30.3437	29.3096	28.0293	27.1651	26.0589	24.9534	24.2751	23.3837
390	34.1812	32.3561	31.0518	29.0064	27.9623	26.7189	25.973	24.8687	23.8029	23.225	22.3469
391	32.6281	30.8249	29.6301	27.6962	26.6822	25.4769	24.7751	23.7228	22.7421	22.1886	21.3326
392	31.0961	29.3463	28.2934	26.3705	25.4227	24.3362	23.6023	22.6227	21.709	21.1837	20.3372

393	29.6202	27.899	26.9433	25.0887	24.2204	23.2139	22.4909	21.5991	20.731	20.176	19.3742
394	28.1545	26.541	25.6788	23.9243	23.0934	22.1192	21.4344	20.6201	19.8347	19.1961	18.4807
395	26.7753	25.2313	24.485	22.7931	21.9982	21.0435	20.4097	19.6126	18.9314	18.2498	17.6067
396	25.4418	23.993	23.295	21.6919	20.8753	20.0055	19.3844	18.6923	17.989	17.3772	16.7852
397	24.1579	22.7591	22.062	20.6122	19.7942	18.9843	18.3864	17.7933	17.048	16.5361	16.0381
398	22.927	21.629	20.9095	19.5875	18.7562	17.9985	17.4262	16.8725	16.176	15.7361	15.2728
399	21.7767	20.4939	19.7592	18.5566	17.7746	17.02	16.5407	15.9945	15.3175	14.9492	14.5021
400	20.6472	19.4255	18.7172	17.5696	16.8507	16.141	15.6917	15.175	14.4894	14.2141	13.7746
401	19.5497	18.3838	17.723	16.6841	16.0305	15.2725	14.8968	14.3843	13.7233	13.519	13.0678
402	18.6134	17.4405	16.8486	15.84	15.2514	14.5046	14.1538	13.6941	13.0527	12.8383	12.3573
403	17.7253	16.5301	16.0609	15.0746	14.5366	13.8307	13.4845	13.0586	12.4354	12.2611	11.7235
404	16.9024	15.7329	15.3378	14.3391	13.9017	13.2397	12.8608	12.4363	11.8536	11.7394	11.184
405	16.1929	15.0427	14.669	13.7196	13.3277	12.6878	12.2922	11.9465	11.3534	11.2706	10.7451
406	15.5907	14.4568	14.1097	13.1358	12.7784	12.2410	11.8111	11.4989	10.9376	10.8076	10.3364
407	14.9895	13.9612	13.6131	12.6492	12.3253	11.7899	11.3809	11.0455	10.5871	10.4441	10.0098
408	14.5351	13.5641	13.166	12.2691	11.9812	11.4104	11.0559	10.6947	10.241	10.0654	9.73553
409	14.2147	13.2381	12.8981	11.9676	11.645	11.1066	10.7897	10.4397	10.0012	9.77145	9.48815
410	13.9360	12.9466	12.6111	11.7091	11.3756	10.8740	10.5664	10.1797	9.76561	9.51239	9.27181
411	13.7200	12.7132	12.3802	11.5153	11.1776	10.6532	10.3636	9.97116	9.55219	9.35234	9.12069
412	13.5551	12.5372	12.2057	11.3267	11.0128	10.5000	10.2062	9.82545	9.37853	9.17767	8.9582
413	13.3971	12.3646	12.0252	11.1222	10.8113	10.3624	10.0329	9.6766	9.28933	9.04737	8.79123
414	13.2089	12.2418	11.8384	11.0045	10.6448	10.2306	9.87391	9.57323	9.16014	8.94294	8.66062
415	13.0691	12.1256	11.7440	10.9013	10.4845	10.0909	9.72802	9.46288	9.04369	8.80922	8.53239
416	12.9764	11.9803	11.6485	10.7703	10.3242	9.98285	9.63616	9.34185	8.94664	8.65755	8.40964
417	12.8530	11.8261	11.5206	10.6805	10.1741	9.84018	9.57275	9.2394	8.83887	8.53725	8.29197
418	12.7051	11.7057	11.4089	10.5813	10.0675	9.68941	9.4756	9.1248	8.71668	8.41943	8.21168
419	12.5695	11.561	11.3015	10.4501	9.9662	9.56622	9.36189	8.97923	8.58428	8.28735	8.12485
420	12.4463	11.429	11.1622	10.2946	9.86865	9.44748	9.24813	8.83147	8.47637	8.17035	8.01212
421	12.2762	11.301	10.9887	10.1428	9.75407	9.31855	9.11656	8.70536	8.36486	8.08033	7.87159
422	12.1305	11.1617	10.8226	9.98969	9.64276	9.1975	8.95589	8.53806	8.24598	7.95417	7.72128
423	12.0074	11.0045	10.6581	9.82461	9.52477	9.0564	8.79352	8.34173	8.06988	7.7923	7.56222
424	11.8292	10.8112	10.4771	9.60859	9.34391	8.89087	8.62744	8.1862	7.92335	7.64251	7.41223
425	11.5902	10.5994	10.2659	9.41272	9.13296	8.70177	8.48336	8.04836	7.75387	7.4745	7.23874
426	11.3733	10.3742	10.054	9.23685	8.96823	8.49925	8.28804	7.84045	7.57118	7.29074	7.06073
427	11.1388	10.1763	9.85777	9.00569	8.76499	8.29711	8.07791	7.67379	7.38445	7.11134	6.92402
428	10.8257	9.93983	9.66736	8.78424	8.55307	8.09059	7.88117	7.50314	7.227	6.94677	6.77039
429	10.608	9.69638	9.45975	8.62241	8.38802	7.89128	7.70911	7.32147	7.0442	6.77421	6.62979
430	10.4143	9.4934	9.26372	8.43812	8.20515	7.73221	7.52385	7.11784	6.88551	6.63534	6.49395
431	10.2183	9.32394	9.05492	8.24976	8.00896	7.60134	7.35362	6.99611	6.72241	6.49103	6.36999

432	10.0168	9.12751	8.86947	8.12619	7.83223	7.48154	7.20271	6.84477	6.57033	6.38354	6.23228
433	9.86315	8.93033	8.67741	7.98911	7.68014	7.37165	7.09213	6.73346	6.44882	6.31431	6.11209
434	9.69888	8.79531	8.51048	7.86079	7.56857	7.28686	6.99044	6.61788	6.37383	6.23947	5.98536
435	9.55467	8.64094	8.37081	7.77103	7.48719	7.19185	6.89105	6.53561	6.28615	6.16481	5.90875
436	9.37268	8.51419	8.28182	7.67619	7.40455	7.07297	6.82363	6.45416	6.21304	6.10007	5.85311
437	9.21447	8.3972	8.18076	7.55682	7.34516	6.9812	6.75772	6.39868	6.14218	6.04429	5.79489
438	9.10005	8.31454	8.09027	7.48204	7.28564	6.93584	6.67943	6.3497	6.08276	5.95281	5.76795
439	9.00594	8.23158	8.00493	7.39174	7.20356	6.8633	6.60497	6.29709	6.01744	5.88252	5.73769
440	8.92558	8.14839	7.93502	7.3126	7.13244	6.80936	6.54111	6.26353	5.97875	5.8492	5.70073
441	8.86422	8.052	7.86479	7.25747	7.08359	6.78671	6.47499	6.21917	5.96895	5.83464	5.69928
442	8.78942	7.981	7.80707	7.21545	7.02268	6.746	6.45171	6.19857	5.96405	5.83944	5.70925
443	8.7148	7.92347	7.76992	7.17348	6.99106	6.72035	6.44971	6.1964	5.98764	5.85387	5.71094
444	8.61644	7.89591	7.71969	7.14964	6.95557	6.70916	6.42542	6.17041	5.97999	5.85319	5.74907
445	8.51734	7.86402	7.66856	7.11707	6.93966	6.68817	6.429	6.15316	5.99057	5.85605	5.78889
446	8.4879	7.85847	7.63035	7.0845	6.93792	6.6681	6.46771	6.18515	6.00056	5.83174	5.81321
447	8.45011	7.8281	7.60831	7.09318	6.96308	6.69207	6.48995	6.19483	6.02134	5.82695	5.83891
448	8.45851	7.80048	7.57952	7.1125	6.97894	6.69723	6.51098	6.20845	6.03663	5.83941	5.88608
449	8.45528	7.78271	7.58914	7.1171	7.01458	6.70504	6.52142	6.25596	6.08179	5.88091	5.88453
450	8.4301	7.789	7.62099	7.13131	7.03067	6.73891	6.54429	6.30534	6.14127	5.91907	5.91483

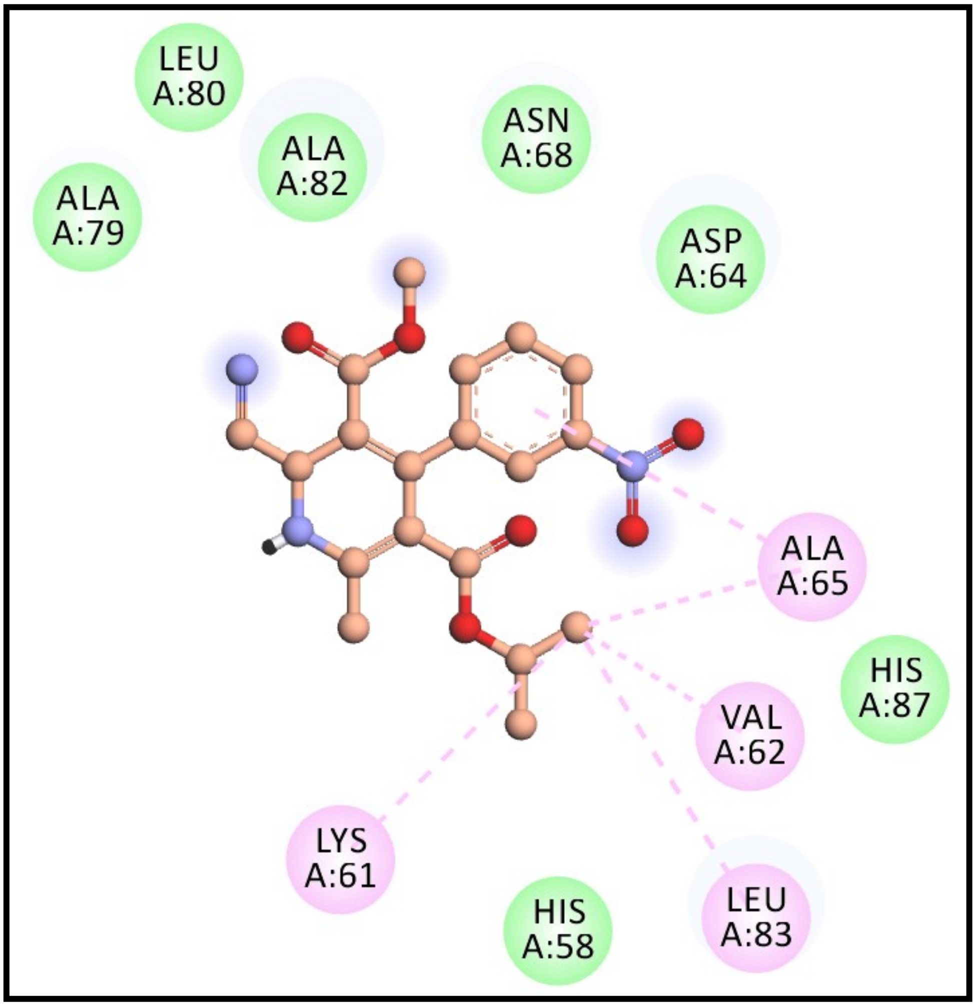

Supplementary Figure 1: Molecular docking of Hb and Nilvadipine using InstaDock software. The binding of Nilvadipine at the same binding site confirmed the reproducibility of the docking protocol used in the study, and validation of the Hb's binding site.

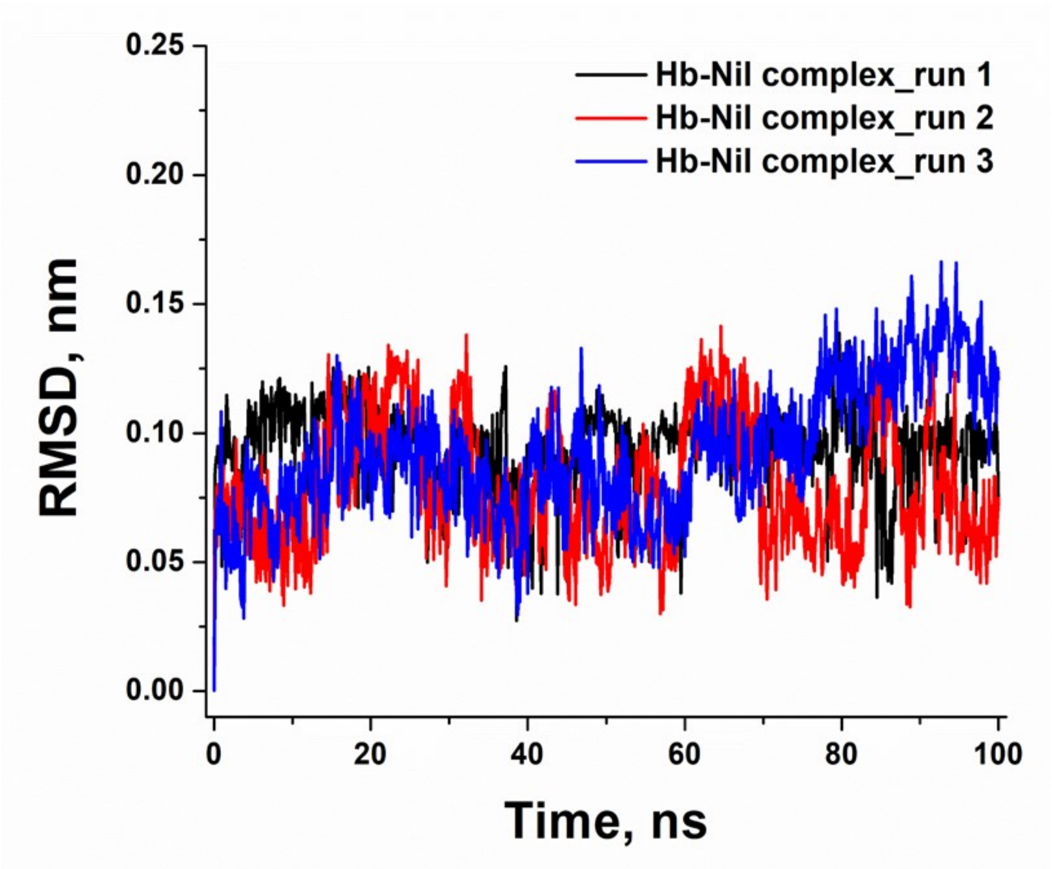

Supplementary Figure 2: Variation in root mean square deviation (RMSD) of Hb in the presence of Nilvadipine. The experiments were performed in triplicates.

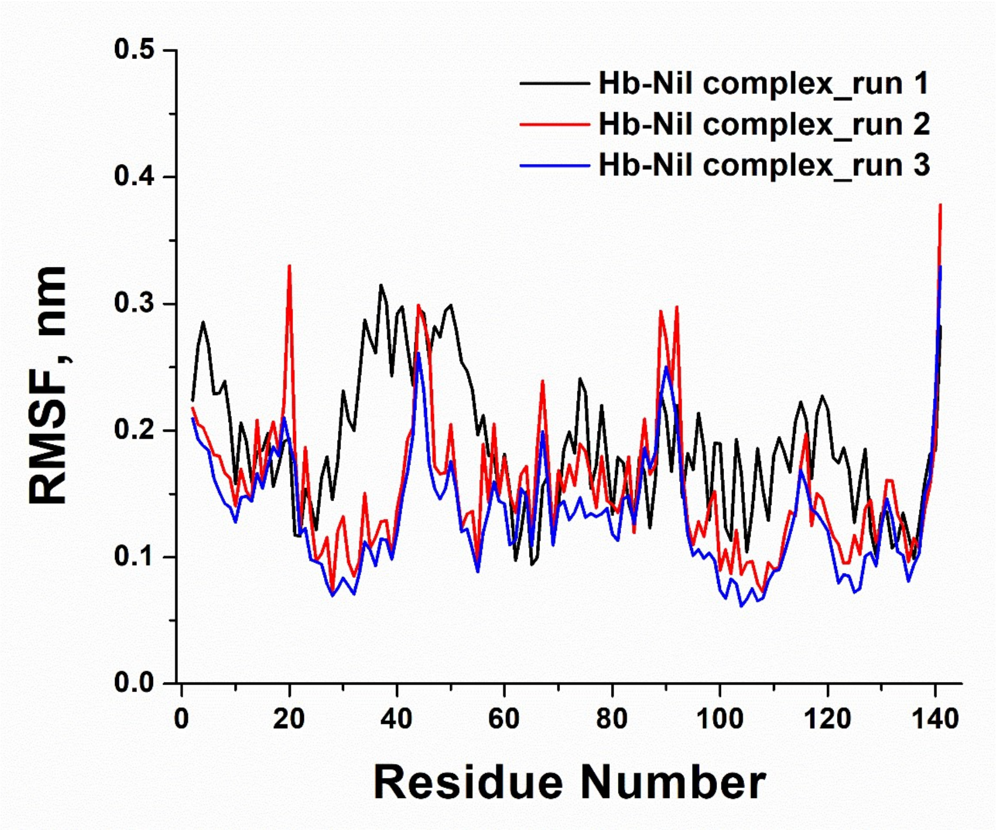

Supplementary Figure 3: Variation in root mean square fluctuation (RMSF) of Hb in the presence of Nilvadipine. The experiments were performed in triplicates.

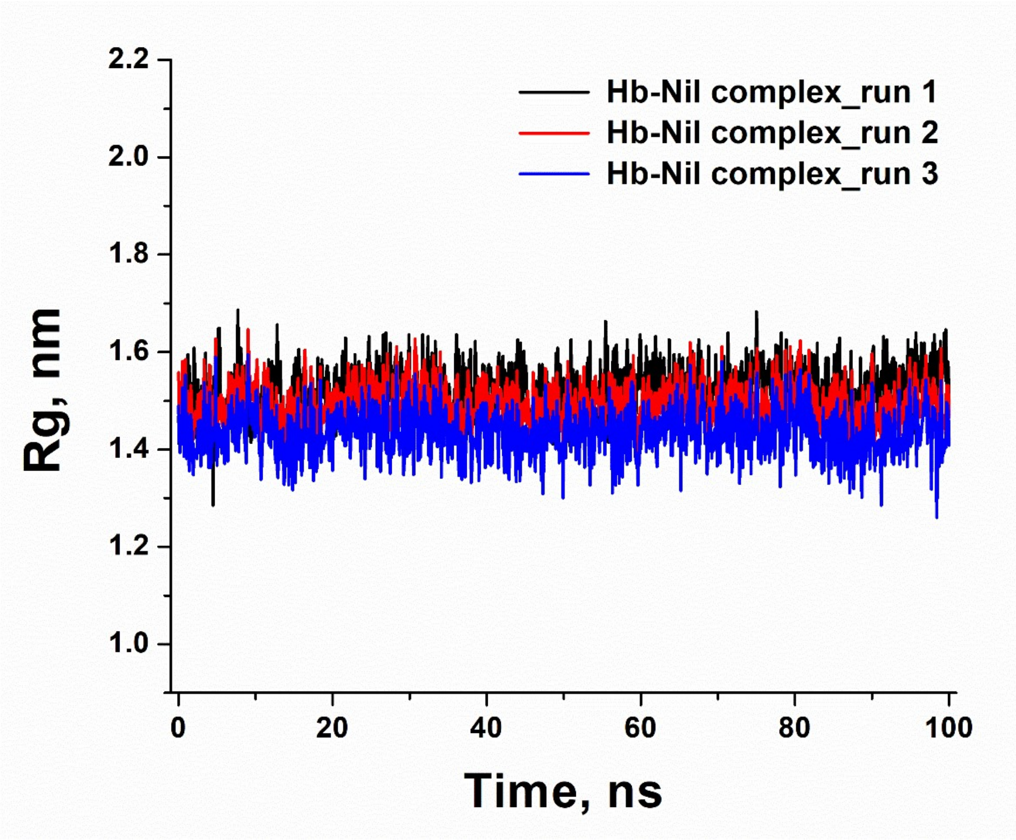

Supplementary Figure 4: Variation in radius of gyration (Rg) of Hb in the presence of Nilvadipine. The experiments were performed in triplicates.

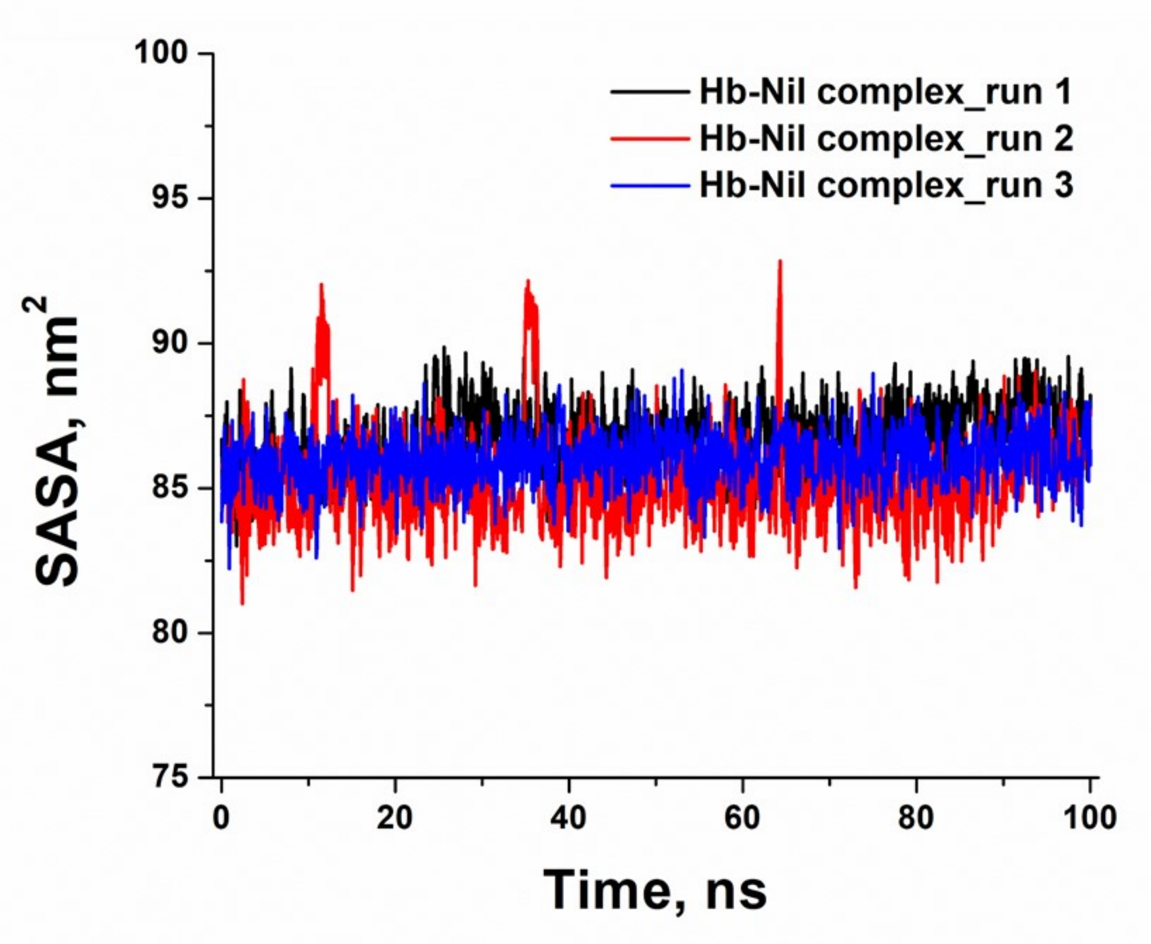

Supplementary Figure 5: Variation in solvent accessible surface area (SASA) of Hb in the presence of Nilvadipine. The experiments were performed in triplicates.

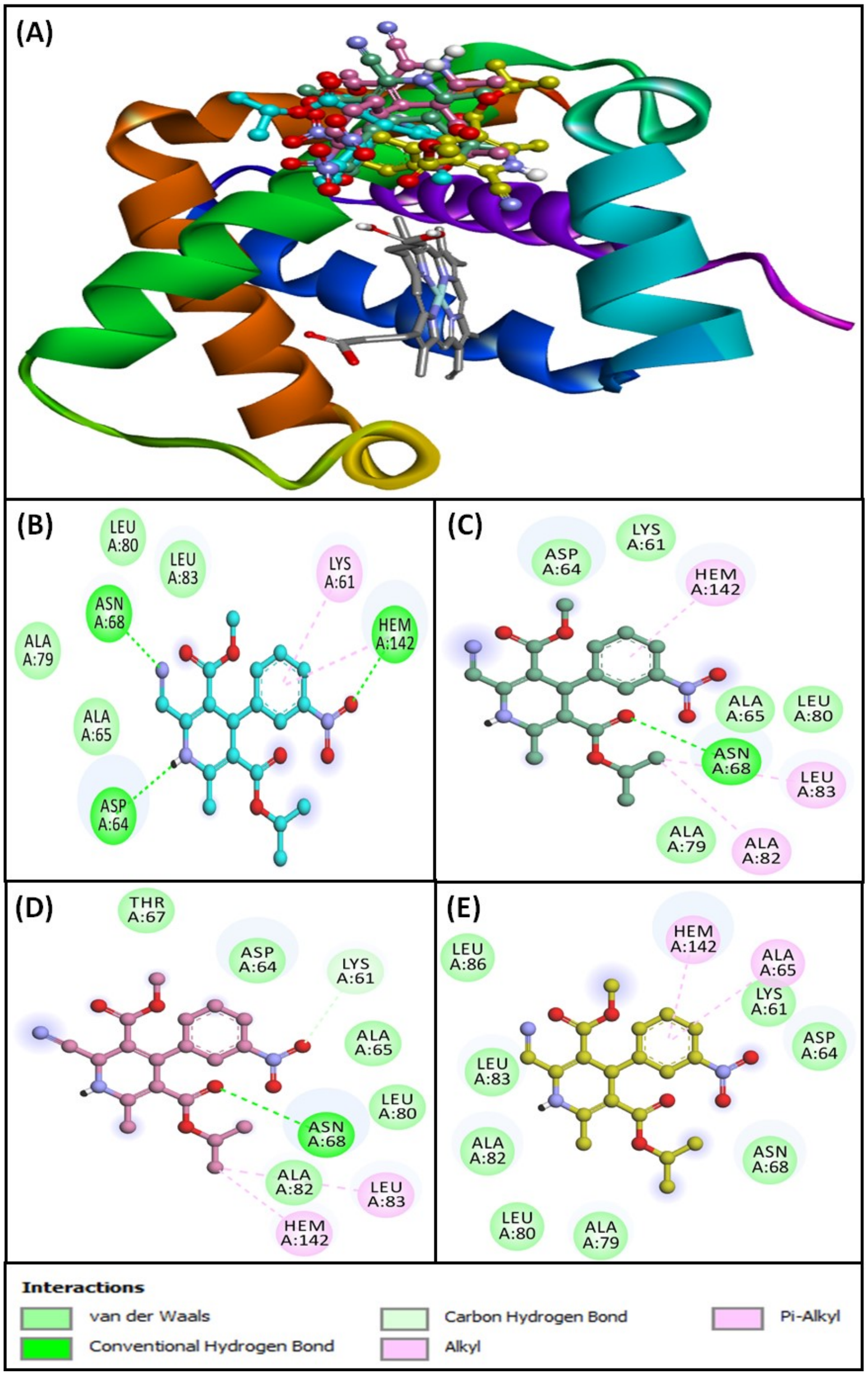

Supplementary Figure 6: Post- MD simulation clustering analysis of the interaction between Hb and Nilvadipine. Clustering was performed using gmx cluster tool of GROMACS with an RMSD cut off 0.2 nm. (A) Representative of different clusters are shown as ball and stick models, (B) Cluster 1, (C) Cluster 2, (D) Cluster 3, and (E) Cluster 4.

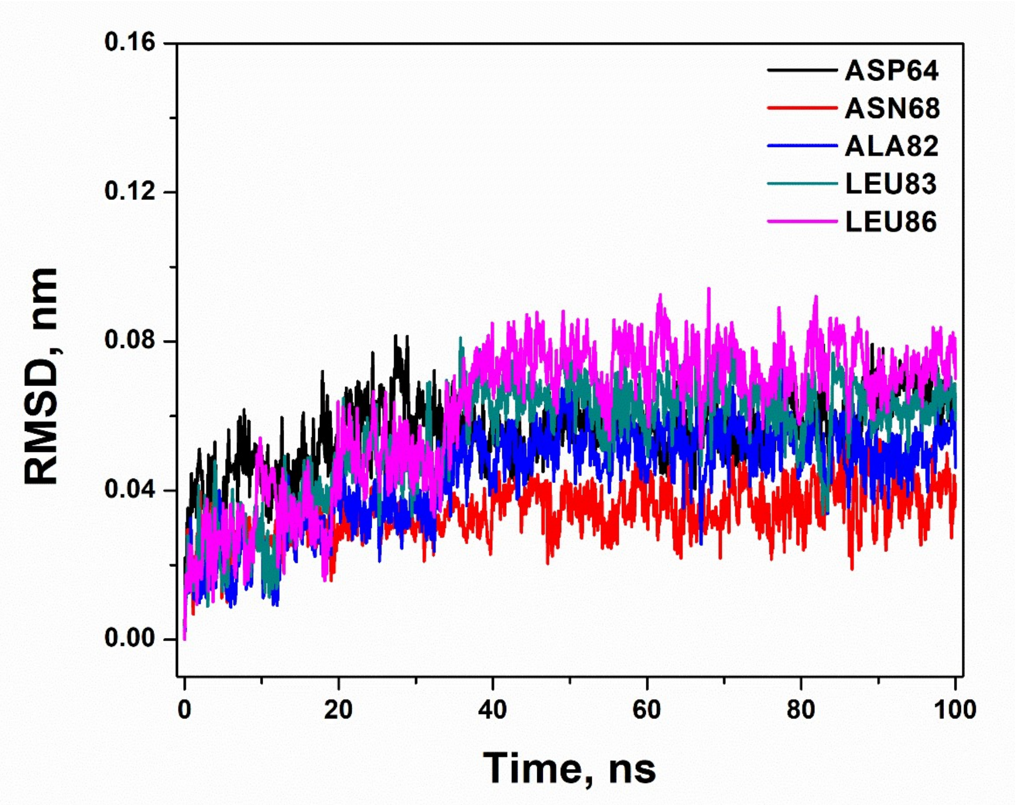

Supplementary Figure 7: Root mean square deviation (RMSD) of key Hb residues (ASP64, ASN68, ALA82, LEU83, and LEU86) interaction with Nilvadipine.
